# Enhanced chromatin accessibility contributes to X chromosome dosage compensation in mammals

**DOI:** 10.1186/s13059-021-02518-5

**Published:** 2021-11-01

**Authors:** Irene Talon, Adrian Janiszewski, Bart Theeuwes, Thomas Lefevre, Juan Song, Greet Bervoets, Lotte Vanheer, Natalie De Geest, Suresh Poovathingal, Ryan Allsop, Jean-Christophe Marine, Florian Rambow, Thierry Voet, Vincent Pasque

**Affiliations:** 1 Department of Development and Regeneration, Laboratory of Cellular Reprogramming and Epigenetic Regulation, KU Leuven – University of Leuven, Herestraat 49, 3000 Leuven, Belgium; 2 KU Leuven Institute for Single Cell Omics (LISCO), 3000 Leuven, Belgium; 3 Leuven Stem Cell Institute (SCIL), 3000 Leuven, Belgium; 4 Laboratory of Reproductive Genomics, Centre for Human Genetics, KU Leuven, 3000 Leuven, Belgium; 5 Laboratory for Molecular Cancer Biology, VIB Center for Cancer Biology, VIB, 3000 Leuven, Belgium; 6 Department of Oncology, Laboratory for Molecular Cancer Biology, KU Leuven, 3000 Leuven, Belgium; 7 VIB-KU Leuven Center for Brain & Disease Research, 3000 Leuven, Belgium

**Keywords:** X chromosome upregulation, X chromosome reactivation, Chromatin accessibility, Gene regulatory networks, iPSC reprogramming, Gene dosage compensation, X chromosome inactivation

## Abstract

**Background:**

Precise gene dosage of the X chromosomes is critical for normal development and cellular function. In mice, XX female somatic cells show transcriptional X chromosome upregulation of their single active X chromosome, while the other X chromosome is inactive. Moreover, the inactive X chromosome is reactivated during development in the inner cell mass and in germ cells through X chromosome reactivation, which can be studied in vitro by reprogramming of somatic cells to pluripotency. How chromatin processes and gene regulatory networks evolved to regulate X chromosome dosage in the somatic state and during X chromosome reactivation remains unclear.

**Results:**

Using genome-wide approaches, allele-specific ATAC-seq and single-cell RNA-seq, in female embryonic fibroblasts and during reprogramming to pluripotency, we show that chromatin accessibility on the upregulated mammalian active X chromosome is increased compared to autosomes. We further show that increased accessibility on the active X chromosome is erased by reprogramming, accompanied by erasure of transcriptional X chromosome upregulation and the loss of increased transcriptional burst frequency. In addition, we characterize gene regulatory networks during reprogramming and X chromosome reactivation, revealing changes in regulatory states. Our data show that ZFP42/REX1, a pluripotency-associated gene that evolved specifically in placental mammals, targets multiple X-linked genes, suggesting an evolutionary link between ZFP42/REX1, X chromosome reactivation, and pluripotency.

**Conclusions:**

Our data reveal the existence of intrinsic compensatory mechanisms that involve modulation of chromatin accessibility to counteract X-to-Autosome gene dosage imbalances caused by evolutionary or in vitro X chromosome loss and X chromosome inactivation in mammalian cells.

**Supplementary Information:**

The online version contains supplementary material available at 10.1186/s13059-021-02518-5.

## Background

Chromatin states vary across cell types [1]. Dynamic changes in nucleosome occupancy, chromatin post-translational modifications and transcription factor (TF) binding to *cis*-regulatory elements in the genome often generate different chromatin accessibility states [2–4]. Changes in the chromatin landscape also contribute to transcriptional processes, gene dosage regulation, and inherited gene silencing [5–7]. A powerful paradigm to study these processes in mammals is X chromosome dosage compensation [8, 9].

To balance for X chromosome differences between female XX and male XY cells, placental mammals have evolved a system in which dosage compensation is achieved by random X chromosome inactivation (XCI) of one of the two X chromosomes during early female embryogenesis [10–14]. This way, only one X chromosome is active in both female and male cells. In addition to XCI, both sexes upregulate the remaining active X chromosome (Xa), in a process known as X chromosome upregulation (XCU), which resolves dosage imbalance between the sole Xa and diploid autosomal gene expression [15–21]. XCU has been reported in several placental mammals including mice [18, 22], marmosets (non-human primates) [23], and humans [18], and in non-placental mammals such as marsupials [14]. Despite advances, the molecular processes underlying the evolution of XCU in mammals remain unclear.

XCI and XCU are developmentally regulated processes [20, 24]. In early mouse embryos, the long non-coding RNA *Xist* initiates XCI by recruiting protein complexes that induce chromosome-wide silencing in *cis* [25–27]. Most genes are subject to XCI with the exception of a small category of genes termed escapee genes [28, 29]. For most genes, silencing in somatic cells is stable even in the absence of *Xist* [30–32]*.* Recently, however, a subset of “*XIST*-dependent” genes in human somatic cells have been reported, where *XIST* is needed to maintain gene silencing [33].

In mice and marsupials, XCU is initiated within the first 3–4 days of development, when imprinted XCI is also initiated in females. XCU is also observed on the sole Xa in males [14, 24, 34–36]. During mouse development, XCI and XCU are both erased in vivo in the naive epiblast, then re-established upon epiblast differentiation and concomitant random XCI [24]. Transcriptional upregulation on the Xa in female cells is thought to be dependent on XCI and has been proposed to be mediated by an increase in transcriptional burst frequency and increased mRNA half-life [22, 24, 37]. Moreover, the hyperactive Xa has been reported to be enriched for active histone modifications, Serine 5 phosphorylated RNA Polymerase II and the histone variant H2A.Z [19, 37]. However, whether chromatin accessibility is enhanced on the upregulated Xa in placental mammals in order to mediate its upregulation is unknown. In addition, how the number of active X chromosomes (Xa’s) in a cell influences chromatin accessibility of the X chromosome relative to autosomes is also unclear.

Chromosome-wide gene silencing from the inactive X chromosome (Xi) in mammalian cells is erased in a process known as X chromosome reactivation (XCR), which has emerged as a paradigm for studying chromatin, gene regulation, development, pluripotency, and reprogramming [38, 39]. In mice, humans and marsupials, XCR takes place in vivo in female primordial germ cells (PGCs) [35, 40, 41], and in the naive mouse epiblast, with the exception of marsupials where XCR does not take place in the epiblast [14]. Recent work also revealed that reactivation of a set of genes from the Xi takes place in human female lymphocytes and in diseases including systemic lupus erythematosus and COVID-19 infection [33, 42]. Therefore, understanding XCR may provide insights into sex-biased diseases in placental mammals. Chromosome-wide XCR can be induced and modeled in vitro using reprogramming of somatic cells into induced pluripotent stem cells (iPSCs) [43]. XCR involves silencing of *Xist*, erasure of repressive chromatin modifications, and chromosome-wide transcriptional reactivation [40, 43–48]. Both in vivo and in vitro, XCR takes place with gene-specific temporal kinetics [47–49]. A small category of genes reactivates early, but this reactivation is restricted to 25% of the iPSC levels [48]. These genes are located in a separate chromatin compartment on the Xi and closer to genes that escape XCI (escapees) [47, 48]. Moreover, chromatin regions in this compartment, which are genomically closer to biallelically accessible regions, also reacquired biallelic chromatin accessibility earlier than other regions during reprogramming [48]. However, whether these observations are due to the analysis of bulk data is not known. Indeed, the precise transcriptional dynamics of XCR at single-cell level and with allelic resolution during iPSC reprogramming have not been defined. How XCR is accompanied by changes in chromatin accessibility during iPSC reprogramming is incompletely understood.

Pluripotency TFs have recently been implicated as factors mediating chromosome-wide XCR [39, 47, 49]. Pluripotency is strongly linked to XCR in mice and humans but not in marsupials, suggesting that placental mammals have evolved molecular mechanisms to couple naive pluripotency with XCR [14, 50]. Indeed, a robust pluripotency gene regulatory network (GRN) leads to *Xist* repression [45, 51]. However, how pluripotency induction during reprogramming leads to a decrease in *Xist* expression followed by XCR remains unclear [39]. In addition, although *Xist* silencing is required for XCR, it is not sufficient [43, 52]. Therefore, additional events beyond *Xist* loss, perhaps including TFs, may be needed to induce XCR. Still, how the pluripotency GRN has evolved in placental mammals to be coupled with XCR and which TFs might play a role in the reversal of chromatin silencing during XCR and after *Xist* is silenced is unclear. Moreover, changes in GRN activity during iPSC reprogramming remain to be comprehensively defined.

Here, we have used allele-specific assay for transposase-accessible chromatin using sequencing (ATAC-seq) to assess chromatin accessibility on the X chromosomes in female somatic cells, during iPSC reprogramming and in male and female mouse embryonic stem cells (mESCs). We found that the upregulated Xa in somatic cells displays enhanced chromatin accessibility relative to autosomes, which we also found on the Xa from male but not female mESCs. Intriguingly, enhanced Xa chromatin accessibility is reversed when the Xi reacquired accessibility during iPSC reprogramming. These results suggest that in placental mammals, increased chromatin accessibility might underlie XCU. Moreover, we followed the temporal transcriptional dynamics of XCU and XCR during iPSC reprogramming with allele-specific single-cell RNA-seq (scRNA-seq). We observed that XCU erasure, which we term X chromosome downregulation (XCD), takes place in parallel with the induction of XCR in cells undergoing reprogramming and involves changes in transcriptional burst frequency. In addition, we found that X chromosome loss in XX iPSCs re-established XCU, and, also involved changes in transcriptional burst frequency, indicating that cells can sense and adapt X chromosome dosage independently of sex and differentiation cues. Additionally, we reconstructed the gene regulatory programs underlying reprogramming to iPSCs to investigate the coupling of XCR to GRN changes. We identified candidate TFs that link pluripotency to XCR, such as ZFP42, which evolved only in placental mammals together with X chromosome dosage compensation in the pluripotent state. Our results support a model where XCR may be coordinated at multiple levels including direct targeting of regulatory elements on the X chromosome, concomitant with stepwise reacquisition of chromatin accessibility. Altogether, our results show how X chromosome dosage compensation in mammals is linked with dynamic changes in chromatin accessibility and GRNs.

## Results

### The single mouse active X chromosome shows enhanced chromatin accessibility

**Fig. 1:**
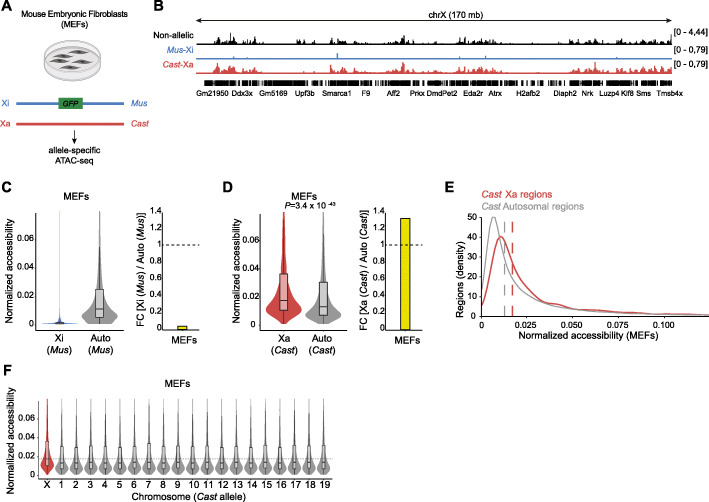
Enhanced chromatin accessibility of the Xa in Female MEFs. **A** Schematic representation of the experimental design used to study allele-specific chromatin accessibility changes on the X chromosomes in female MEFs with ATAC-sequencing. **B** ATAC-seq signals for non-allelic (black) and allelic (*Mus*, blue and *Cast*, red) chromatin accessibility of the entire X chromosome. **C** Violin plot combined with boxplot showing normalized accessibility of the Xi chromosome and the median of all the autosomes (left) and fold change (FC) of the median normalized accessibility of the Xi relative to the autosomes (right) for the *Mus* allele in female MEFs. A Wilcoxon rank-sum test was used for significance testing. **D** Violin plot combined with boxplot showing normalized accessibility of the Xa chromosome and the median of all the autosomes (left) and fold change (FC) of the median normalized accessibility of the Xa relative to the autosomes (right) for the *Cast* allele in female MEFs. A Wilcoxon rank-sum test was used for significance testing. **E** Density plot with X-linked (red) and all autosomal (grey) regions from the *Cast* allele showing normalized accessibility in female MEFs. **F** Violin plot combined with boxplot showing normalized accessibility of the X chromosome and all the autosomes for the *Cast* allele. The dashed line indicates the median accessibility on the X-*Cast*

To examine chromatin on the X chromosomes, we measured chromatin accessibility in female mouse embryonic fibroblasts (MEFs) with a maternal Xi, through allele resolution ATAC-seq (Fig. 1A, B, Additional file 1: Fig. S1A). These cells were derived from a hybrid cross between *Musculus* females carrying an X-linked GFP reporter and *Castaneus* males (hereafter *Mus* and *Cast*, respectively), enabling allele-specific analyses [27, 29, 47, 49, 53]. In addition, we sorted GFP-negative cells to ensure that the *Mus* X chromosome allele is inactivated (Xi-*Mus* and Xa*-Cast*). As expected, the Xi displayed a chromosome-wide reduction in median peak chromatin accessibility compared with autosomes (Fig. 1C) [5, 54]. Unexpectedly, however, the Xa showed enhanced chromatin accessibility relative to autosomes (Fig. 1D–F). Specifically, the Xa was globally 1.33 times more accessible than the median of all autosomes on the same allele (Fig. 1D, right). These results suggest that a 1.33-fold increase in chromatin accessibility contributes to X chromosome to autosome gene dosage compensation, which is in line with what is observed at the transcriptional level [22]. We confirmed enhanced chromatin accessibility on the Xa by measuring allelic median peak chromatin accessibility and allelic X-to-autosomes (X/A) accessibility ratios (Additional file 1: Fig. S1B-C). Enhanced chromatin accessibility on the Xa is consistent with XCU and the enrichment of active histone modifications on the Xa in MEFs [19]. The increase of global chromatin accessibility on the Xa is also consistent with an increased in Xa chromatin accessibility in *Drosophila*, but has not yet been described in mammals where sex chromosomes evolved independently [55]. Thus, the Xa of female differentiated cells in mice is associated with chromatin hyperaccessibility.

### Enhanced chromatin accessibility on the active X chromosome is reversed by reprogramming to pluripotency

**Fig. 2:**
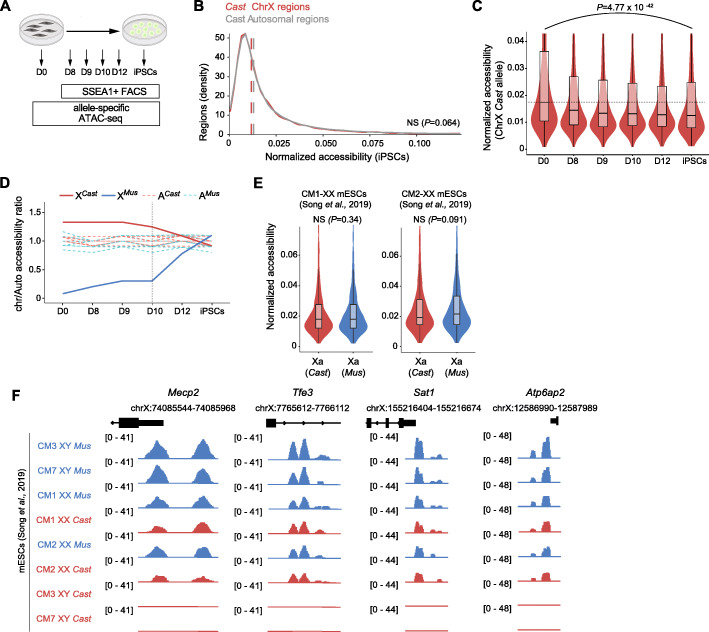
Chromatin Hyperaccessibility on the Xa is reversed during reprogramming to pluripotency. **A** Schematic representation of the experimental design used to study allele-specific chromatin accessibility changes on the X chromosomes during reprogramming of female MEFs to iPSCs. **B** Density plot with X-linked (red) and all autosomal (grey) regions from the *Cast* allele showing normalized accessibility in female iPSCs. A Wilcoxon rank-sum test was used for significance testing. **C** Violin plot combined with boxplot showing X chromosome normalized accessibility ratio for the *Cast* allele during reprogramming. Dotted line marks day 0 normalized accessibility. A Wilcoxon rank-sum test was used for significance testing. **D** Chromosome to autosome accessibility ratio for the *Cast* (red) allele and the *Mus* allele (blue). Continuous lines indicate the X chromosome to autosome ratio and dotted lines indicate individual autosome to all other autosomes ratio. Vertical dotted line indicates day 10 changes. **E** Violin plot combined with boxplot showing X chromosomes normalized accessibility for the Xa-*Cast* and Xa-*Mus* alleles in XX mESC lines (CM1 and CM2) from [56]. A Wilcoxon rank-sum test was used for significance testing. **F** ATAC-seq signals for allelic chromatin accessibility of Mecp2, Tfe3, Sat1, and Atp6ap2 transcript regions in XY and XX mESCs. The Mus allele is shown in blue and the Cast allele is shown in red. Reanalysis of data from [56]

Unlike differentiation which induces XCI, reprogramming to pluripotency induces XCR. However, how reprogramming to pluripotency and XCR affect chromatin accessibility of the Xa is unknown. To address this, we assessed allele-specific chromatin accessibility during reprogramming and in female XX mouse iPSCs. We reprogrammed Xi-*Mus* Xa-*Cast* MEFs into iPSCs, isolated SSEA1+ reprogramming intermediates at days 8, 9, 10, and 12 as well as iPSCs, and applied allele-specific ATAC-seq (Fig. 2A, Additional file 1: Fig. S2A) [47]. Clustering of autosomal accessibility confirmed genome-wide changes in chromatin accessibility during reprogramming, while genomic tracks showed gains in chromatin accessibility at pluripotency genes (Additional file 1: Fig. S2B-E). To assess chromatin accessibility on the Xa during reprogramming, we performed allele resolution chromatin accessibility analyses. We found that enhanced chromatin accessibility on the Xa in female MEF cells is erased in XX iPSCs (Fig. 2B, Additional file 1: Fig. S2F). In addition, we observed an increase in the number of accessible peaks on the X chromosome during reprogramming (Additional file 1: Fig. S2D), consistent with a gain of accessibility on the Xi. We also observed that female XX mouse iPSCs lack enhanced chromatin accessibility on the Xa by analyzing X chromosome accessibility counts and allelic chromatin accessibility ratios to autosomes during reprogramming (Fig. 2C, D, Additional file 1: Fig. S2G). Furthermore, unlike X chromosomes, autosomal median peak accessibility remained stable throughout reprogramming (Additional file 1: Fig. S2H). Our results suggest that reprogramming to pluripotency erases enhanced chromatin accessibility on the Xa in female cells.

In addition, we investigated the dynamics of enhanced chromatin accessibility erasure on both X chromosomes during reprogramming. Enhanced chromatin accessibility on the Xa was still present at day 8 and day 9 of reprogramming and decreased at day 10 and day 12, but was lost in iPSCs (Fig. 2D). Intriguingly, reacquisition of chromatin accessibility on the other X chromosome allele, the Xi, seemed to take place concomitant with the loss of enhanced chromatin accessibility on the Xa (Fig. 2D). These results suggest that cells sense the number of Xa’s and may adapt chromatin accessibility levels accordingly.

To further test this in pluripotent stem cells, we analyzed published allele-specific ATAC-seq data from two XX mESC lines (females), named CM1 and CM2, and two XY mESC lines (males), named CM3 and CM7, resulting from a hybrid cross between *Mus* females and *Cast* males [56]. While both X chromosome alleles in female mESCs showed the same level of accessibility as autosomes, resembling the X chromosome state of XX iPSCs (Fig. 2E, F), we found that chromatin accessibility on the X-*Mus* chromosome of male XY mESCs was increased 1.4 fold over that of autosomes (Additional file 1: Fig. S2I-J). This was observed in most of the accessible regions, whereas several regions in male mESCs did not show increased accessibility (Additional file 1: Fig. S2K). Specifically, among 1263 X-linked regions shared between the female CM2 mESCs and male CM7 mESCs, 64% increased accessibility by 10–25% in the male X*-Mus* compared to female X*-Mus*, while 13% regions showed increased accessibility by less than 10% and 22% of regions did not show increased accessibility in the male X*-Mus* compared to the female counterpart (Additional file 2: Table S1). These results suggest that enhanced Xa accessibility is due to increased accessibility at a large and specific subset of X-linked chromatin regions. Altogether, these data also suggest that enhanced chromatin accessibility on the Xa is induced when only one Xa is present or active in a diploid cell, independently of the parental allele origin, and is reversed when the Xi re-gains accessibility during XCR.

### Allele-specific scRNA-seq of iPSC reprogramming establishes the transcriptional dynamics of XCR at single-cell resolution

**Fig. 3:**
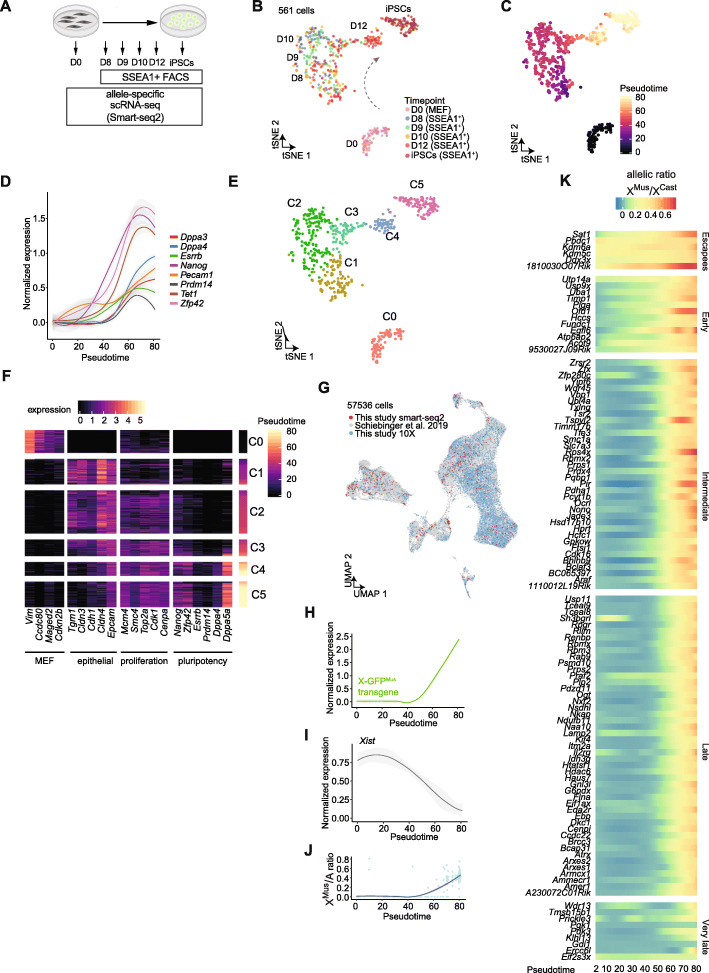
Allele-specific scRNA-seq analysis of XCR during iPSC reprogramming. **A** Experimental design to study allele-specific single-cell gene expression changes during reprogramming to iPSCs. **B** tSNE of gene expression levels (log2-transformed normalized counts) of the reprogramming dataset (*n* = 561 cells) colored by isolation time point. Each dot represents a cell. **C** tSNE visualization with cells colored by pseudotime along the reprogramming trajectory. Each dot represents a cell. **D** Normalized expression levels of representative pluripotency markers plotted along pseudotime. The fitted line was derived using the loess function. Grey areas represent the 95% confidence interval. **E** tSNE visualization with cells colored by the different reprogramming clusters. Each dot represents a cell. **F** Normalized expression of genes from selected cellular signatures in single cells during iPSC reprogramming. **G** UMAP of single-cell gene expression colored by dataset. Each dot represents a cell. **H** Expression of X-GFP transgene plotted along pseudotime trajectory. Fitted line derived using loess function. Grey areas around the fitted line represent the 95% confidence interval. **I** Expression of *Xist* plotted along pseudotime trajectory. Fitted line derived using loess function. The grey area around the fitted line represents the 95% confidence interval. **J** Ratio between expression from X*-Mus* allele and average autosomal expression in each single cell and modelled along pseudotime. The fitted line was derived using the loess function. The grey area around the fitted line represents the 95% confidence interval. **K** De novo kinetics of XCR reconstructed using loess regression to model the X-*Mus* to *Cast* allelic ratio calculated in each cell as a function of pseudotime for each gene. *K*-means clustering was used to classify by reactivation kinetics. Gene expression levels were normalized to library size (number of total counts per library) in **D**, **F**, **H**, and **I**

We next set out to examine the temporal transcriptional changes taking place on the Xi during XCR in iPSC reprogramming. Transcriptional kinetics during XCR and iPSC reprogramming have been described in bulk populations or without allele resolution analyses [43, 47, 48, 57–59]. Yet, the exact timing of XCR at allele-specific single-cell resolution is not known. To determine the dynamics of transcriptional changes during XCR and iPSC reprogramming, we performed allele-specific Smart-seq2 scRNA-seq (Fig. 3A). We analyzed Xi-*Mus* Xa-*Cast* MEFs, SSEA1+ reprogramming intermediates, and iPSCs. t-Distributed Stochastic Neighbor Embedding (tSNE) arranged cells into several groups that reflected reprogramming progression (Fig. 3B, C, Additional file 1: Fig. S3A). As expected, we detected the activation of pluripotency-associated genes including the early activation of *Pecam1* and *Zfp42*, followed by *Nanog*, *Tet1*, and *Esrrb*, and others later in reprogramming such as *Dppa3*/*4* and *Prdm14* (Fig. 3D).

We were interested to know whether other hallmarks of reprogramming are also present in the datasets. We therefore grouped cells into 6 clusters, ordered cells by reprogramming pseudotime, and analyzed gene expression. We detected a gradual switch from a MEF-specific state to a mesenchymal-to-epithelial transition state (C1 cells), which is one of the first step towards successful reprogramming (Fig. 3E, F) [60, 61] and is followed by a proliferative state and the activation of early pluripotency markers such as *Zfp42*, *Pecam1*, and *Nanog* (C2 cells, Fig. 3D–F). This wave of transcriptional activation is followed by the acquisition of additional pluripotency genes, including *Dppa4* and *Dppa5a* (C4 and C5 cells), as expected [58, 59, 62–64] (Fig. 3E, F, Additional file 1: Fig. S3B). Therefore, scRNA-seq analysis recapitulates the transcriptional changes that take place during iPSC reprogramming. To further determine the transcriptional identity of cells in this study, we integrated our data with additional datasets (Additional file 1: Fig. S3C). To do this, we performed 10X Genomics scRNA-seq analysis of day 16 unsorted reprogramming populations using two independent transgenic reprogrammable stem cell cassette (STEMCCA) mouse systems [43, 65]. We also used a published single-cell atlas of iPSC reprogramming with *Mus* genetic background, which does not enable allele resolution analyses [59]. Integration revealed that our reprogramming intermediates cluster together with the corresponding time points from the reference datasets (Fig. 3G, Additional file 1: Fig. S3D). We also performed gene signature enrichment analysis to map the activity of six distinct signatures: MEF, epithelial, pluripotent, neural, senescent, and trophoblast onto the integrated dataset (Additional file 1: Fig. S3E-F). We found that reprogramming intermediates activated mostly epithelial and pluripotent signatures while only very few cells exhibited neural and senescent identities (Additional file 1: Fig. S3G-H). This further confirms that our reprogramming intermediate cells represent cells undergoing reprogramming. In sum, we generated single-cell transcriptomes that map cell fate conversion from MEFs to iPSCs and recapitulate findings from previous studies, but unlike previous datasets, our new data also enabled allele-specific analyses (see below).

Using our allele resolution single-cell transcriptomic data during conversion of polymorphic MEFs into iPSCs, we quantified allelic gene expression for 439 X-linked genes. Both *Mus*-derived X-linked GFP expression and the Xi to autosome allelic ratio (X*-Mus*/A) revealed chromosome-wide Xi reactivation during pluripotency induction after the initiation of *Xist* RNA silencing (Fig. 3H–J). These results are in agreement with late XCR during iPSC and the requirement for *Xist* silencing for XCR [43, 45–48, 59]. However, previous studies also suggested that different genes reactivate at different times during XCR, with a small category of “early” reactivating genes [47–49]. To define the transcriptional kinetics of XCR after resolving cellular heterogeneity, we determined the Xi/Xa allelic ratio of X-linked genes as a function of pseudotime and classified genes by reactivation kinetics using *k*-means clustering (Fig. 3K). This revealed the presence of 5 distinct groups of genes which we named escapees, early, intermediate, late, and very late reactivating genes, in line with previous findings by bulk RNA-seq [47, 48]. In addition, we detected new escapee genes including *Ddx3x*, *Sat1*, and facultative escapee *1810030O07Rik* as the most highly activated escapee gene (Fig. 3K). We also detected new early genes including *Uba1*, *Timp1*, *Ofd1*, *Fundc1*, and *Egfl6*. Several early genes including *Usp9x*, *Atp6ap2*, and *Acot9* were also identified as early genes in a previous study using bulk RNA-seq [47] (Fig. 3K, Additional file 1: Fig. S3I). *Xist* silencing seemed to be initiated early, around the time when early genes initiate reactivation (Additional file 1: Fig. S3J). However, even at the single-cell level, the complete reactivation of early reactivated genes is achieved only late in reprogramming, concomitant with activation of the pluripotency GRN and *Xist* silencing (Fig. 3K, Additional file 1: Fig. S3J) [43, 45, 46, 48, 57]. Nearly all intermediate and late genes seemed to reactivate around the same time. Finally, we identified *Ercc6l* as a new late reactivated gene. We also detected genes, including *Wdr13* and *Prickl3*, that were reactivated, then became inactivated then reactivated again. Taken together, our results establish the precise transcriptional dynamics of XCR at allele-specific single-cell resolution during iPSC reprogramming. These data also clarify the different sensitivities of individual X-linked genes for reactivation during iPSC reprogramming. Early genes partially reactivate early and are then reactivated to full levels later, together with chromosome-wide reactivation of most Xi-linked genes. However, several genes can also reactivate very late.

### XCU erasure is coupled to XCR during pluripotency induction

**Fig. 4:**
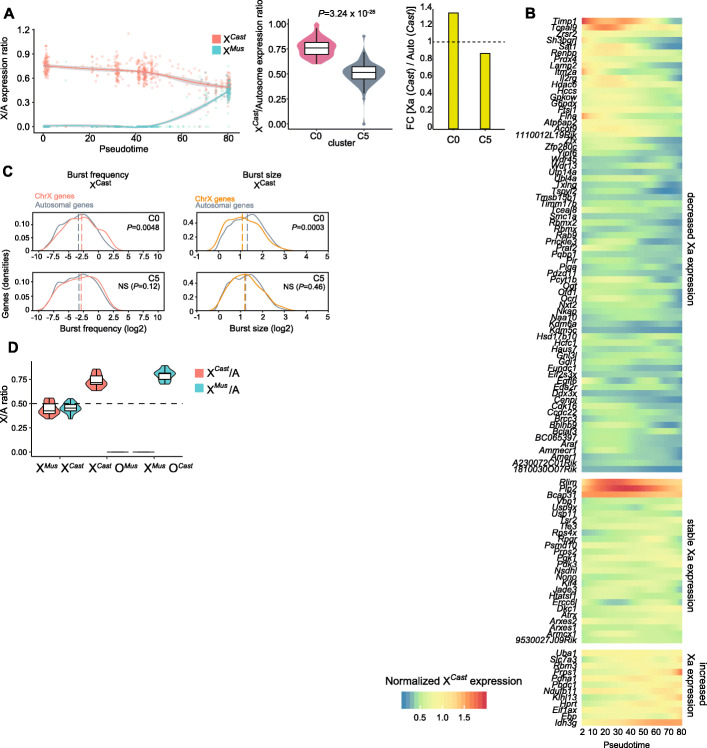
Erasure of XCU during iPSC reprogramming and XCU induction following aneuploidy of the X chromosome. **A** Expression ratio of the X*-Mus* allele (blue) and X-*Cast* allele (red) to autosomes (X/A) as a function of reprogramming pseudotime trajectory (left) and between cells in cluster 0 and 5 defined in Fig. 3E (right). A Wilcoxon rank-sum test used for significance testing. **B** Kinetics of XCU erasure on a per gene basis reconstructed using loess regression to measure X-*Cast* normalized expression in each cell as a function of pseudotime. The resulting inferred values at equal pseudotime intervals are classified by expression pattern using *k*-means clustering. **C** Distribution of burst frequency (left) and burst size of autosomal and X-linked genes on the *Cast* allele in cells from cluster 0 and 5 defined in Fig. 3E. A Wilcoxon rank-sum test used for significance testing. **D** X/A ratio for *Cast* (red) and *Mus* (blue) alleles. The dashed line indicates the expected X/A ratio when both X chromosome alleles are expressed like autosomal alleles

Given that XCR takes place during reprogramming, increasing X-linked gene transcripts from the Xi, and the loss of enhanced chromatin accessibility on the Xa, we investigated how transcriptional dosage of the Xa is mediated during reprogramming. We calculated X/A ratios for each allele separately, X*-Mus*/A and X*-Cast*/A, along the reprogramming pseudotime. Unexpectedly, we found that XCD, the erasure of XCU on the Xa of MEFs, took place during iPSC reprogramming. The median expression of X-*Cast* genes was 1.35 fold higher than that of autosomes in MEFs (C0), while it was below that of autosomes in iPSCs (C5) (Fig. 4A, Additional file 1: Fig. S4A). We confirmed XCD by analyzing median allelic expression (Additional file 1: Fig. S4B). A reanalysis of bulk RNA-seq data from our previous study also confirmed XCD [47, 66]. Unexpectedly, XCD is most pronounced when XCR takes place on the other allele. XCD is also consistent with loss of Xa chromatin hyperaccessibility (Fig. 2). Taken together, the analysis shows that XCD takes place during reprogramming, mostly concomitant with XCR and loss of enhanced chromatin accessibility on the Xa, indicating that X-linked gene dosage compensation may be tightly regulated during reprogramming to iPSCs.

Next, we examined the timing of XCD on a per gene basis. We found that the majority of genes decrease in expression from the Xa during reprogramming, with a notable decrease when XCR takes place around pseudotime 50 (Fig. 4B, decreased Xa expression group). For instance, *Acot9*, *Atp6a2*, and *Sat1* genes that are reactivated early on the other allele (Xi), were initially highly expressed on the Xa and their expression decreased around the time XCR took place. In addition, we also observed genes on the Xa that exhibit distinct behavior with no change in expression or an increase in expression during reprogramming (stable and increased Xa expression groups) (Fig. 4B). Several X-linked genes have a higher total gene expression (Xi+Xa) in iPSCs relative to MEFs, probably due to the difference in cell identity and their X chromosome states. In consequence, both alleles of those X-linked genes increase their expression during reprogramming (Additional file 1: Fig. S4C). Taken together, these results reveal distinct expression dynamics for different genes on the Xa, with a majority of genes accounting for XCD.

Previous studies proposed that XCU in somatic cells is mediated by increased transcriptional burst frequency (how often a pulse of transcripts production occurs) [22, 24]. We asked whether XCD during reprogramming could be mediated by changes in transcriptional burst frequency and/or burst size (number of transcripts generated per transcriptional pulse). We inferred transcriptional burst parameters with the two-state model of transcription, in which switching between ON and OFF states of a gene occurs at rates of *k*_on_ and *k*_off_, and transcription only occurs in the ON state with a rate of *k*_syn_ [67]. This model provides allele-specific estimates of burst frequency (*k*_on_) and burst size (*k*_syn_/*k*_off_) [22, 24, 67]. We found that burst frequency was indeed significantly increased (1.19 fold increase) on the Xa relative to autosomes in MEFs (Fig. 4C, left, cluster C0, Additional file 1: Fig. S4D), in line with previous reports in mouse primary fibroblasts [22]. However, we also observed lowered burst size (0.82 fold decrease) on the Xa relative to autosomes in MEFs (Fig. 4C, right, Additional file 1: Fig. S4D), which is not concordant with previous reports in mouse primary fibroblasts [22]. Interestingly, we found that differences in both burst frequency and burst size between Xa and autosomes were lost after reprogramming to iPSCs, consistent with XCD (Fig. 4C, right, Additional file 1: Fig. S4D). In summary, XCD takes place during reprogramming to iPSCs and involves a decrease in transcriptional burst frequency and an increase in burst size.

### X chromosome aneuploidies are dosage compensated by XCU in iPSCs

Previous studies have reported that one X chromosome can be lost after prolonged culture of iPSCs [56, 68], reflecting a well-known phenomenon in embryonic stem cells (ESCs) [56, 68–73] and a condition termed Turner syndrome, the only viable monosomy in humans [74]. We used this property to test whether XCD depends on the presence of two Xa’s. We performed additional scRNA-seq of female XO iPSCs that had lost either the *Cast* or the *Mus* X chromosome as judged by loss of biallelic escapee gene expression (Additional file 1: Fig. S4E), as well as control XX iPSCs. We next investigated the effect of X chromosome loss on gene expression dosage from the remaining X chromosome allele in iPSCs. The two X chromosomes of XX iPSCs were expressed at levels similar to autosomes. Unexpectedly, however, XO cells upregulated their sole Xa, regardless of which X chromosome became aneuploid (Fig. 4D). We also observed that the absolute expression from a single Xa was significantly higher in XO cells compared to each Xa of XX cells (Additional file 1: Fig. S4F). Thus, the loss of one of the two X chromosomes in XX iPSCs seems to be dosage compensated by XCU on the remaining X chromosome.

Moreover, we asked if X chromosome loss results in changes in transcriptional burst frequency and size in iPSCs. We found that XX iPSCs showed similar levels of burst frequency and size on both alleles of the X chromosome and on autosomes (Additional file 1: Fig. S4G, top row). Cells which lost an X chromosome (X-*Cast* O-*Mus*) showed a significant increase in burst frequency of X-linked genes and no decrease in burst size in agreement with XCU and in agreement with a previous study [22] (Additional file 1: Fig. S4G, middle row). Burst frequency was also increased when the opposite X chromosome allele is lost. Specifically, the cells which lost the *Cast* X chromosome (X-*Mus* O-*Cast*) displayed increased burst frequency of X-linked genes (Additional file 1: Fig. S4G, bottom row), and a significant decrease in burst size compared to autosomes (Additional file 1: Fig. S4G, bottom row), which recapitulates our findings of transcriptional burst in MEFs (Fig. 4C). As expected, X chromosome loss was apparent from the depletion of reads from the aneuploid X chromosome (Additional file 1: Fig. S4G). Therefore, XO cells undergo changes in transcriptional burst on their sole X chromosome that might contribute to XCU.

Collectively, the results show that XCU is erased concomitant with XCR during reprogramming, then reinstated after X chromosome loss in female iPSCs and accompanied by an increase in transcriptional burst frequency. X chromosome dosage sensing and compensation are thus intrinsic properties of cells both in the differentiated and undifferentiated states.

### Chromatin changes during XCR

**Fig. 5:**
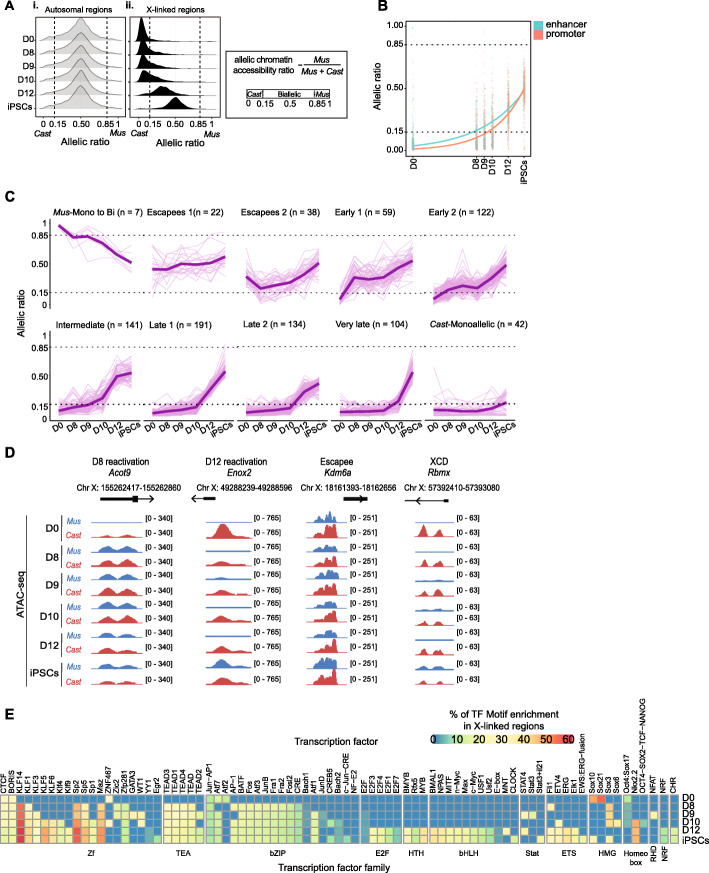
The Xi reacquires chromatin accessibility during reprogramming to iPSCs. **A** Density plots showing regions with (i) allelic autosomal and (ii) X-linked chromatin accessibility ratios across different reprogramming time points from day 0 to iPSCs. Allelic chromatin accessibility ratios were calculated by dividing maternal read counts by total reads (*Mus*/*Mus* + *Cast*). The number of informative regions is 750. **B** Allelic ratio (*Mus*/*Mus* + *Cast*) of all X-linked annotated enhancer (blue) and promoter (red) regions that become biallelically accessible during iPSC reprogramming. **C** Allelic ratio (*Mus*/*Mus* + *Cast*) of all informative X-linked regions (*n* = 750) at each time point of iPSC reprogramming. Regions were grouped by *k*-means clustering. The bold line represents the average allelic ratio at each time point for regions within each cluster. **D** ATAC-seq tracks for allelic chromatin accessibility at promoter regions of representative X-linked gene regions that become biallelically accessible at different time points (day 0, day 12, escapees, and XCD) during reprogramming. The parental origin of the accessible allele is indicated in red for *Cast* and in blue for *Mus*. **E** Enrichment of TF motifs in X-linked biallelic accessible regions at different time points during reprogramming. Only significant enrichments (*p* value ≤ 0.05) are shown. The color gradient represents the percentage of regions with enriched motifs in the indicated group over 50,000 random background genome regions

To determine the region-specific temporal dynamics of chromatin accessibility changes during XCR, we used our allele-specific chromatin accessibility data during reprogramming. During XCI, the Xi becomes globally inaccessible [5], yet chromosome-wide inaccessibility is reversed by reprogramming of neural progenitor cells derived in vitro [48]. However, exactly when chromosome-wide chromatin accessibility is re-established during XCR in our system, in which XCI is induced in vivo, has remained unclear. To answer this question, we first calculated chromatin accessibility ratios for autosomal and X-linked regions. On autosomes, biallelic chromatin accessibility was maintained throughout reprogramming (Fig. 5Ai, Additional file 1: Fig. S5A-B). Unlike autosomes, we observed a chromosome-wide transition from monoallelic to biallelic chromatin accessibility on the X chromosomes (Fig. 5Aii). In addition, we annotated X-linked promoter and enhancer regions based on a combination of chromatin marks using ChromHMM [75] (Additional file 3: Table S2). This revealed that reacquisition of chromosome-wide chromatin accessibility during XCR took place late during reprogramming, both at enhancers and promoters, with slightly earlier opening of enhancers compared to promoters (Fig. 5B). Altogether, XCR provides a unique example of chromosome-wide reestablishment of chromatin accessibility.

We next set out to define the detailed kinetics of chromatin accessibility on the Xi during XCR. We used *k*-means clustering to cluster X-linked regions based on changes in allelic chromatin accessibility ratios. This analysis revealed sets of specific regions, on the Xi, that acquire accessibility at different times during reprogramming (Fig. 5C). We grouped 10 clusters into 7 distinct categories based on chromatin accessibility dynamics: inaccessible regions on the Xi-*Mus* that become biallelically accessible at day 8 (early 1 and 2 clusters), at day 9 (intermediate cluster), day 10 (late 1 and late 2 clusters), and at day 12 (very late cluster) during XCR, regions that remain biallelically accessible throughout reprogramming (escapees 1 and escapees 2 clusters), regions accessible from the *Mus* allele that become biallelically accessible (*Mus*-mono to bi cluster), and regions that are only accessible from the *Cast* allele (*Cast*-monoallelic cluster) (Fig. 5C, Additional file 1: Fig. S5C). The region-specific temporal changes in chromatin accessibility were also seen at gene promoters including *Acot9*, which became accessible by day 8 of reprogramming and *Enox2*, that was biallelically accessible after day 12 of reprogramming (Fig. 5D). Our results show that there is a region-specific temporal order of changes in chromatin accessibility on the Xi during XCR.

To better understand what might influence the dynamics of chromatin accessibility during XCR, we asked if the acquisition of chromatin accessibility can be explained by the genomic distance to pre-existing biallelically accessible regions. During XCR, early reactivated genes reside closer to escapee genes [47], but the relationship between chromatin opening and escapee regions is not clear. We measured the distance of chromatin regions from each cluster to the closest biallelically accessible regions in MEFs. We found that chromatin regions that open earlier on the Xi during XCR (early 1 and 2) are, on average, closer to biallelically accessible regions in MEFs (Additional file 1: Fig. S5D). However, not all regions close to escapee regions opened early during XCR. The findings are in line with a recent study in neural progenitor cells [48] and suggest that the distance to biallelically accessible regions is a predictor of chromatin opening kinetics during reprogramming independently of starting cell types. Altogether, these results show that chromatin regions that become accessible first during XCR tend to be closer to pre-established accessible regions. Thus, reprogramming to iPSCs induces acquisition of chromatin accessibility at specific sites on the Xi which is subsequently propagated to other regulatory elements including enhancers and promoters.

To understand how changes in chromatin accessibility relate to transcriptional activation, we associated chromatin regions to genes and compared to our previously reported kinetics of transcriptional Xi reactivation (Additional file 1: Fig. S5E-F) [47]. We found a partial overlap between chromatin accessibility and transcriptional activation kinetics (Additional file 1: Fig. S5F). Altogether, these data indicated that chromatin accessibility kinetics partially correlate with transcriptional kinetics during XCR. In summary, we defined the chromosome-wide temporal hierarchy of chromatin events on the Xi during XCR.

### Relationship between TFs and XCR chromatin accessibility dynamics

To gain insights into putative TFs that might drive XCR, we analyzed the TF motifs associated with *cis*-regulatory elements that become gradually accessible during XCR. This revealed that chromatin regions that open on the Xi at different times are enriched for distinct sets of TF motifs, including motifs for the binding of the non-pluripotent TFs KLF14, SP2, and MAZ as most enriched motifs from reprogramming day 8, present in 50–60% of the analyzed X-linked regions (Fig. 5E). We also found the enrichment of motifs for the pluripotent TFs KLF4 (from reprogramming day 10) and c-MYC (from day 12) (Fig. 5E) and SOX2, the latter was enriched specifically in X-linked enhancer regions (Additional file 1: Fig. S5G). However, these pluripotency-associated TF motifs were only found in 20–30% of the X-linked regions that become biallelically accessible during reprogramming. Moreover, we examined published ChIP-seq data for several pluripotency TFs and the p300 histone acetyltransferase in male mESCs [76] and found a higher enrichment of OCT4, SOX2, KLF4, c-MYC, ESRRB, PRDM14, NANOG, and p300 binding at regions that become biallelically accessible at day 8 of reprogramming (early 1 and 2) compared with regions that become biallelic later during reprogramming (Additional file 1: Fig. S5H). To validate our findings, we used our previously published ChIP-qPCR data of OCT4 binding at promoter regions of X-linked genes that reactivate early (*Acot9* and *Sat1*) during reprogramming (Additional file 1: Fig. S5I) [47]. These data confirm that OCT4 can bind to chromatin regions that become accessible early in reprogramming. We also previously showed a higher degree of OCT4 binding at these sites at day 8 of reprogramming compared to day 15 [47], further supporting a link between pluripotency TFs and accessibility of putative regulatory elements during XCR. Collectively, we mapped the accessibility landscape of XCR and identified a catalog of TFs that are potentially implicated in XCR.

### Gene regulatory networks during iPSC reprogramming

A comprehensive understanding of how GRNs are reconfigured during reprogramming to iPSCs and linked with XCR is lacking. To understand how the pluripotency GRN is linked with XCR in placental mammals, we first explored the GRN changes that take place during iPSC reprogramming.

**Fig. 6:**
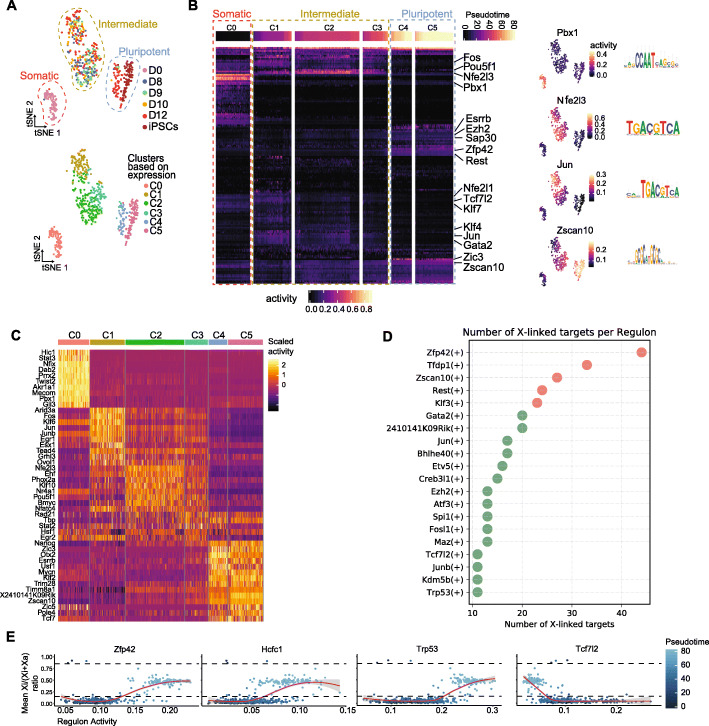
Single-cell gene regulatory network inference reveals candidate regulators of XCR during iPSC reprogramming. **A** tSNE visualizations of single-cell clustering based on regulon activity. Each dot represents a cell. Top tSNE: colors indicate isolation time point (pink = day 0, blue = day 8, green = day 9, yellow = day 10, red = day 12, and brown = iPSCs) with cell states marked with dashed lines (red = somatic, yellow = intermediate and blue = pluripotent). Bottom tSNE: colors indicate graph-based clustering classification (red = C0, yellow = C1, green = C2, light blue = C3, dark blue = C4, and pink = C5). Clustering based on regulon activity was performed with SCENIC on Smart-seq2 dataset. The activity of 311 regulons in total was quantified. **B** Heatmap of regulon activity ordered by cell state and pseudotime (*x*-axis). Dashed lines indicate cell states (red = somatic, yellow = intermediate, and blue = pluripotent). Selected regulons are indicated in *Y*-axis and regulon activity of selected regulons is shown in tSNEs (right) with the corresponding motif. **C** Heatmap with regulon activity of regulons with the highest specificity for each cluster (C0–C5). **D** Plot with the number of X-linked targets per regulon. Top 20 regulons with the most X-linked targets are shown. **E** Relationship between regulon activity and XCR. Four regulons are displayed with the highest coefficients of logistic regression model of X-linked genes allelic ratio on regulon activity. Fitted line on the plot was generated using loess function. The grey areas represent the 95% confidence interval

We reconstructed the GRNs active during iPSC reprogramming by applying single-cell regulatory network inference and clustering (SCENIC) to our scRNA-seq data [77, 78]. SCENIC first identifies TF targets based on gene co-expression with TFs in the same cell, then the list of targets is filtered to keep only the targets which contain a binding motif for a given TF [77]. The outcome is a list of regulons, where each regulon is a collection of predicted gene targets for a given TF. SCENIC identified 311 regulons active during reprogramming (Additional file 4: Table S3). Based on the activity of target genes, these regulons are predicted to be potentially involved in reprogramming. Clustering cells based on TF (regulon) activity revealed 3 distinct states: somatic, intermediate, and pluripotent (Fig. 6A). Somatic regulons such as Pbx1 are rapidly turned off, before the activation of the earliest pluripotency markers, while key pluripotency-associated regulons, such as Zscan10, become activated later (Fig. 6B). Interestingly, we identified a set of regulons specific for the intermediate reprogramming state, which transiently activates TFs related to e.g., AP1 or Wnt signaling pathways (JUN and TCF7L2 factors, respectively, previously implicated in iPSC reprogramming [79, 80]) (Fig. 6B, Additional file 1: Fig. S6A). Together, these results show that iPSC reprogramming is characterized by dynamic changes in regulatory activity.

Next, we set out to define which TFs potentially drive cell identity within each of the previously defined clusters. We ordered regulons based on regulatory activity within each cluster (Additional file 1: Fig. S6B). Regulon activity can serve as a useful metric of TF activity and refers to the proportion of expressed genes in the signature (regulon) and their relative expression values compared to the other genes within the cell. Cluster C0 has a high regulatory activity for somatic TFs including Tead1. Cluster C1 cells already show regulatory activity for several pluripotency-associated regulons such as Nanog, which eventually became one of the most active regulons in clusters C3–C5 (Additional file 1: Fig. S6B). These results suggest a progressive increase in pluripotency TF regulatory activity during reprogramming and are consistent with previous reports of early regulatory activity for Nanog during factor-induced reprogramming [59, 64, 81]. We also identified several other TFs whose regulatory activity is potentially important for cell identity during reprogramming, such as Tead4, which has recently been proposed to regulate chromatin accessibility during reprogramming of human cells to iPSCs (Fig. 6C, Additional file 1: Fig. S6B) [82]. In addition, we used the Wilcoxon rank-sum test to establish a list of regulons with activity specific to each cluster. This analysis revealed that intermediate state clusters 1–3 exhibit transient activity of many regulons such as Jun, Junb, and Nfe2l3 (Fig. 6B, C). Furthermore, these analyses show that the regulatory activity of pluripotency regulons of cluster 4 and cluster 5 is similar (Fig. 6B, C), suggesting that the iPSC GRN is faithfully established following the activation of late pluripotency markers. Altogether, these findings indicate that GRNs undergo a global remodeling during reprogramming to iPSCs and transit through a distinct, intermediate regulatory state. Furthermore, our analyses revealed a comprehensive collection of putative regulatory targets.

To better understand whether the intermediate regulatory state may promote entrance into pluripotency or is associated with alternative reprogramming outcomes, we performed GRN inference with SCENIC on the 57536 cells of the scRNA-seq atlas of iPSC reprogramming containing data from this study (Smart-seq2 and 10X scRNA-seq) as well as from a previous non-allelic study [59]. This resulted in defining the activity of 499 regulons (Additional file 5: Table S4). Integration followed by Uniform Manifold Approximation and Projection (UMAP) analysis based on regulatory activity showed that regulatory states of the 3 reprogramming systems can be easily aligned across the entire reprogramming trajectory (Additional file 1: Fig. S6C). Next, we monitored the activity of regulons active in the intermediate state across cells which activated previously defined gene signatures. We found that most of the intermediate regulons are activated in cells with epithelial signatures (Additional file 1: Fig. S6D), suggesting that these regulons may be associated with a transient epithelial state along a successful reprogramming trajectory. However, we also identified intermediate-state-specific regulons such as Gata2, Tbp, and Hsf1 which were strongly activated in cells with trophoblast identity or Phox2a regulon specifically active in cells with a neural signature (Additional file 1: Fig. S6D). Therefore, our analyses allowed us to define regulatory states and TFs which might drive either intermediate reprogramming states and terminal, alternative outcomes to neural or trophoblast lineages and provide a resource for further studies of cell fate reprogramming.

### Link between pluripotency GRNs and XCR

The detailed mapping of gene regulatory states during reprogramming enabled us to investigate how the pluripotency GRN is linked to XCR. We leveraged TF-target network interactions to predict TFs that might directly target X-linked genes, and hence might be candidate regulators of XCR. In a first approach, we ranked regulons by the number of X-linked targets (Fig. 6D). This revealed a list of TFs including ZFP42, TFDP1, and ZSCAN10 as top candidates, followed by KLF3, ATF3, and MAZ whose motifs were also enriched in the chromatin regions becoming biallelically accessible during reprogramming (Fig. 5E). We propose that these TFs might be regulators of X-linked gene expression. In a second approach, we set out to test which regulons can be best correlated with XCR. To this end, we performed logistic regression to measure the probability with which regulon activity predicts XCR (Fig. 6E). Ordering regulons by decreasing regression coefficient revealed that the activity of Zfp42, Hcfc1, and Trp53 regulons correlates best with transcriptional reactivation of the Xi (Fig. 6E). Hence, we identified candidate regulators of XCR.

ZFP42 is of particular interest because (1) it has the highest number of predicted X-linked gene targets of all regulons (Fig. 6D), (2) it evolved specifically within placental mammals, together with the evolution of *Xist* in species such as mouse and human where naive pluripotency is linked with the presence of two Xa’s, unlike in marsupials where pluripotency and XCR are uncoupled in the epiblast [14, 83, 84], and (3) ZFP42 has been reported as a repressor of *Xist* and activator of *Tsix* but not yet implicated in XCR [83]. We also found that putative ZFP42 X-linked targets are enriched on the X chromosome relative to autosomes, even after taking gene density into account (Additional file 1: Fig. S6E, Additional file 6: Table S5). Moreover, HCFC1 and TRP53, whose regulon activity is correlated with reprogramming pseudotime and XCR (Fig. 6E), also showed putative X-linked targets enriched on the X chromosome relative to autosomes (Additional file 1: Fig. S6E, Additional file 6: Table S5), contrary to TCF7L1 which has more putative targets enriched on autosomes (Additional file 1: Fig. S6E, Additional file 6: Table S5) and whose regulon activity is anti-correlated with reprogramming pseudotime and XCR (Fig. 6E). In addition, reanalysis of published ZFP42 ChIP-seq data in mESCs [85], together with the gene annotation for regions where ZFP42 was bound, revealed that ZFP42 is bound to approximately 33% of genes on the X chromosome, which further supports our predictions based on GRN analysis (Additional file 1: Fig. S6F). These findings raise the possibility that ZFP42 evolved to couple naive pluripotency with XCR. In addition to ZFP42, we identified additional candidates. We integrated the chromatin accessibility motif enrichment analysis of Xi reactivation with regulon activity during reprogramming (Additional file 1: Fig. S6G). This revealed additional candidate TFs whose motifs are enriched in accessible chromatin and have high regulatory activity, such as KLF3, KLF5, KLF6, TEAD4, and MAZ. In summary, we reconstructed the GRNs of mouse iPSC reprogramming and identified candidate transcriptional regulators of XCR.

## Discussion

**Fig. 7:**
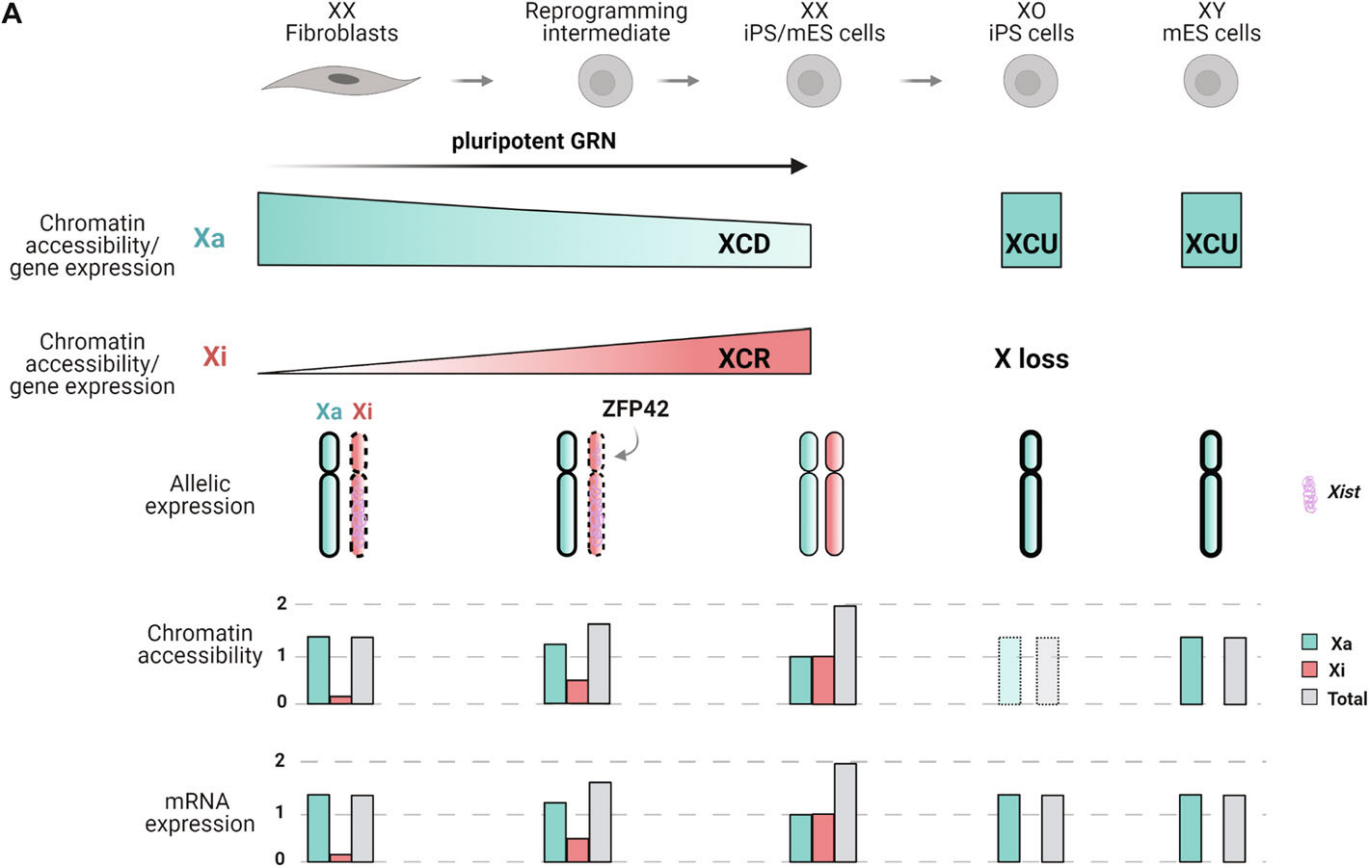
Proposed model of X chromosome dosage compensation during mouse iPSC reprogramming. Scheme of dosage compensation on the Xa and Xi during reprogramming of female fibroblast to iPSCs and consecutive loss of one X (XO). Events taking place on the Xa are marked in green and in red for the Xi. On the somatic state, the Xa is hyperactive and hyperaccessible compared to autosomal levels. During reprogramming, the Xa undergoes XCD and chromatin hyperaccessibility decreases until it reaches the level of autosomes in the pluripotent state. Concurrently, the Xi gains chromatin accessibility and is reactivated with different genes reactivating at different times during reprogramming. From the pluripotent state, one X chromosome can be lost, which induces upregulation of the sole Xa. The doted box shows the predicted accessibility level of the Xa in XO iPSCs

Collectively, we propose a model based on our findings, summarized in Fig. 7, in which we show that dosage compensation in mice results in enhanced chromatin accessibility on the single Xa, which might mediate transcriptional upregulation of the Xa after XCI in somatic cells and after X chromosome loss in iPSCs. We also observed that both enhanced chromatin accessibility and transcriptional upregulation are erased during the induction of pluripotency, concomitantly with XCR, suggesting a dosage sensing mechanism between the two X chromosomes. Moreover, we mapped dynamic changes in the chromatin accessibility landscape during XCR and in combination with scRNA-seq we identified TFs that are putatively involved in XCR. Our analyses revealed that factors such as ZFP42 might have evolved to couple pluripotency with XCR. Altogether, these data illustrate how gradual acquisition of a new GRN during reprogramming of cellular identity is linked with dynamic induction of chromatin accessibility and overcomes stable chromatin silencing on the Xi.

### Enhanced chromatin accessibility on the Xa in mammals

Our study identified enhanced chromatin accessibility on the upregulated Xa in mouse female fibroblasts and male mESCs, but not in XX female iPSCs or mESCs. This suggests that enhanced chromatin accessibility may be linked to XCU to mediate dosage compensation in mammals. Previous studies suggested that several placental mammals, including mice and humans, and also non-placental mammals, such as marsupials, evolved XCU to mediate dosage compensation between one Xa and diploid autosomes [14, 17, 18, 22, 86]. Our results not only strengthen claims of XCU in mammals, but also suggest that enhanced chromatin accessibility contributes to XCU in mice. They may also provide additional evidence for Ohno’s hypothesis that mammalian dosage compensation evolved in two steps: (1) hyperactivation of the X chromosome in both sexes to compensate for gene loss on the Y chromosome, and (2) silencing of one of the two X chromosomes by XCI [86]. In *Drosophila*, the male Xa shows enhanced chromatin accessibility [55]. However, sex chromosomes and dosage compensation evolved independently in *Drosophila* and mammals [18]. Hence, the evolution of sex chromosomes and dosage compensation may have independently evolved dosage compensation mechanisms leading to enhanced Xa chromatin accessibility in different species.

In this study, Xa enhanced chromatin accessibility was detected in two situations. First, on the upregulated Xa in female differentiated cells after XCI, and second on the sole Xa from male mESCs (Fig. 7). Therefore, cells might sense the number of Xa’s present in a cell and induce X chromosome chromatin hyperaccessibility only when one Xa is present. Moreover, XCU has also been observed in humans [17, 18]. Hence, it is possible, and quite likely, that enhanced chromatin accessibility is present in human somatic cells on the single Xa in both males and females. However, additional work is needed to test this hypothesis, which will be facilitated by advances in single-cell epigenomics. Of note, surprisingly, a recent study using single-cell ATAC-seq (scATAC-seq) did not detect enhanced Xa chromatin accessibility in mice [24]. However, detecting Xa enhanced accessibility necessitates to focus on chromatin regions with sufficiently good coverage (see “Methods”). Thus, with sparse scATAC-seq data, grouping cells first and then focusing on regions with sufficient reads (i.e., > 10 reads) might be needed to detect Xa chromatin hyperaccessibility.

During evolution, different species have adopted different strategies to mediate dosage compensation, and several lines of evidence indicate that multiple mechanisms are involved [21, 87]. For mammalian XCU, mechanisms acting both at the transcriptional and posttranscriptional level have been reported including increased Pol II Ser5P binding, increased histone H4K16 acetylation, increased transcriptional bursting, and increased mRNA half-life [19, 22, 37, 88]. Our results suggest that enhanced chromatin accessibility is also involved in mammalian XCU and is consistent with increased H4K16 acetylation on the Xa [37]. Previous studies suggested that not all genes on the X chromosome have the same dosage sensitivity [89]. This agrees with our observation of region-specific enhanced chromatin accessibility on the Xa. Hence, we speculate that specific regulatory elements associated with dosage-sensitive genes are preferentially subject to compensation by enhanced chromatin accessibility. Furthermore, we observed that Xa enhanced chromatin accessibility is erased during reprogramming to female iPSCs. This suggests a sensing mechanism involving trans-factors shared or coordinated between both X chromosomes.

### Kinetics of XCR during iPSC reprogramming

Using allele-specific scRNA-seq we reveal the precise temporal kinetics of XCR. The finding that a subset of genes reactivates early is in line with previous work using bulk RNA-seq [47–49]. *Xist* silencing seems to start early, suggesting that early genes may be sensitive to *Xist* loss (Additional file 1: Fig. S3J). This may be related to the mechanisms of facultative escape because early reactivated genes tend to share genomic and chromatin organization features with escapee genes [47, 48]. The reactivation of early genes may relate to the mechanisms of facultative escape from XCI. Although *Xist* has been long thought to be dispensable for maintenance of XCI, recent studies revealed the presence of subsets of *Xist*-dependent genes in mouse and human somatic cells [32, 33, 90]. While we cannot exclude that the early genes identified in our study do not depend on *Xist* silencing, it is possible that a subset of early genes reactivate just as *Xist* silencing is initiated, and early genes may be more dependent on *Xist* for maintenance of silencing. This is consistent with the cell-type-specific early genes identified during the reprogramming of MEFs and neural progenitor cells [47, 48] and also the cell-type-specific dependencies of *XIST* in human somatic cells [33]. Thus, variable sensitivity to *Xist* could explain the reactivation of early genes and additional experiments are needed to comprehensively determine the impact of *Xist* removal on the kinetics of XCR. Other features such as short distance to escapee genes and 3D organization may also be involved.

The reactivation of early genes could be relevant for diseases. XCR for a subset of genes has recently been reported in patients with autoimmune disease including systemic lupus erythematosus and COVID19 [33]. Moreover, XCR has been proposed as a potential therapeutic target for Rett Syndrome [91]. Understanding XCR will provide new insights for medical purposes.

In addition to early genes, nearly all other genes seem to reactivate nearly at the same time. This is in contrast with a previous study that used bulk RNA-seq [47] and in line with a recent study in neural progenitor cells [48]. The difference can be explained by the use of allele-specific scRNA-seq and the reprogramming system used. The new results suggest that once *Xist* is silenced and the full pluripotency GRN is activated, repressive chromatin marks are lost from the Xi and most genes reactivate along the X chromosome. We did also identify a small category of genes that reactivate late, which may depend on additional modes of regulation, such as HDACs [47].

### Kinetics of XCU and XCD during iPSC reprogramming

In this study, we observed that XCD takes place concomitantly with XCR, which is consistent with XCD in vivo in the naive epiblast and in vitro [24, 66], but in contrast with a reprogramming study in PGCs that observed Xa upregulation after XCR [92]. It would be interesting to also document XCU during PGC development with allele resolution. XCI has been proposed to be linked to the initiation of XCU during female early development [24]. On a genome-wide level, XCD coincides with XCR. Yet, at the gene level at least, XCD and XCR do not appear to be always linearly proportional to each other for all genes during iPSC reprogramming. These results are consistent with XCR of restricted gene sets after *XIST* knockout in somatic cells, without significant change on the Xa [33]. It will be of interest to identify the *cis*-regulatory elements that functionally sense and mediate gene dosage compensation by XCU. The co-occurrence of XCD and XCR during reprogramming suggests that cells possess the intrinsic property of balancing X-linked gene dosage.

XCU was shown to operate via an increase in transcriptional burst frequency [22, 24]. However, it remained an open question whether transcriptional burst frequency is also modulated during XCU erasure. Moreover, it has been proposed that burst frequency is regulated by enhancers [22, 24, 93] which suggests that dosage compensation on Xa and Xi is, at least in part, regulated at the level of enhancers. A key question that remains unanswered is which factors are responsible for sensing X chromosome dosage. One hypothesis could be that XCU is mediated by the limited availability of one or more *trans*-activator, which is distributed over accessible X chromosomes, as recently proposed [94]. This might involve the acetyl transferase MOF and H4K16ac, as suggested by [37]. However, the investigation of the X-specific effects of MOF in mouse is hindered by the lethality of MOF null mutations [95, 96]. Conversely, there might exist an X-linked repressor, which mediates upregulation erasure when expressed from the Xa. Moreover, we also observed a reduced burst size on the Xa compared to the autosomes, which is contrary to what was found in a previous study [22], where autosomes and the upregulated Xa do not differ in transcriptional burst size. However, these different observations could arise due to the lack of unique molecular identifiers (UMIs) in the Smart-seq2 method used in our study. UMIs can help improve inference of transcriptional burst kinetics [67]. Therefore, it would be interesting to use single-cell transcriptomics approaches that use UMIs, such as Smart-seq3, to further investigate this point.

### TFs and XCR

Our previous work, as well as the work of others, suggested that binding of pluripotency TFs to promoters of a subset of X-linked genes might be implicated in XCR [47, 49]. For instance, X-linked genes that reactivate early in the naive epiblast are enriched for c-MYC binding in mESCs [49]. In addition, during iPSC reprogramming, early reactivating genes have been found to be enriched for KLF4 and ESRRB binding in mESCs [47]. PRDM14 has been identified as another TF that might mediate XCR [46]. However, the precise definition of factors that are responsible for initiating and establishing XCR, and the regulatory regions by which they operate has been elusive. Here, we integrated multiple modalities and approaches to identify TFs that might be involved in XCR.

We found ZFP42 as a potential TF which might directly target X-linked genes for reactivation. ZFP42 has already been previously associated with the regulation of *Xist* and *Tsix* in mouse during XCI, but not yet during XCR [83, 85]. In addition, ZFP42 is not present in marsupials, where pluripotency is uncoupled to XCR [14]. We propose a new mechanism by which the pluripotency TF ZFP42 directly targets multiple X-linked genes along the X chromosome for reactivation in addition to targeting the X inactivation center. However, further experiments are required to elucidate the exact role of ZFP42 in XCR.

Secondly, the analysis of TF binding motifs in regions that become accessible at different times revealed TFs that might be involved in XCR. Motifs belonging to the ZF-KLF family, which are known to be important for genome-wide reprogramming [97] where the most enriched on the X chromosome during reprogramming. In addition, this family also appeared as one of the main XCR candidates in our genome-wide GRN analysis. Moreover, we found the YY1 motif enriched from day 12 of reprogramming. Interestingly, ZFP42 is phylogenetically related to YY1, originating from a duplication of YY1 in placental mammals and their motifs share some similarity in their core regions [84]. Moreover, YY1 and ZFP42 have been shown to regulate *Xist* by competing binding to the *Xist 5’* region and activating (YY1) or repressing (ZFP42) *Xist* expression [98]. Allele resolution chromatin immunoprecipitation analyses would be required to further understand the role of TFs in the reversal of gene silencing during XCR.

Altogether, we demonstrate how gradual acquisition of a new GRN during reprogramming of cellular identity is linked with dynamic induction of chromatin accessibility and how it overcomes stable chromatin silencing on the Xi. Our findings pave the way for a better understanding of epigenetic reprogramming, highlighting the central role of chromatin remodeling and TFs in X chromosome dosage compensation and reprogramming.

### Limitations

To study the landscape of chromatin accessibility on the X chromosomes during reprogramming, we used bulk ATAC-seq. This provides good coverage and allele-specific resolution. We were able to confidently detect 750 allele resolution X-linked regions with information at all reprogramming time points to follow dynamics of XCR. However, similarly to transcriptomics, some of the details might be lost by averaging cell populations in bulk and therefore scATAC-seq could provide additional insights.

GRN inference is an exciting approach to gain an understanding of how TF networks might be remodeled during cell fate changes as well as to identify TFs that might be responsible for regulating specific target genes of interest, for instance, X-linked genes. However, currently, these methods still generate a list of putative targets as the actual factor binding data is often not involved in target gene determination. Future studies are required to determine actual TF binding with allele resolution during iPSC reprogramming.

## Methods

### Cell culture

MEFs were cultured in MEF medium [DMEM (Gibco, 41966-554 052) supplemented with 10% (v/v) fetal bovine serum (FBS, Gibco, 10270-106), 1% (v/v) penicillin/streptomycin (P/S, Gibco, 15140-122), 1% (v/v) GlutaMAX (Gibco, 35050-061), 1% (v/v) non-essential amino acids (NEAA, Gibco, 11140-050), and 0.008% (v/v) beta-mercaptoethanol (Sigma-Aldrich, M7522)]. Cells undergoing reprogramming and iPSCs were cultured in mouse ESC medium [KnockOut DMEM (Gibco, 10829-018) supplemented with 15% FBS, 1% P/S 10,000 U/mL, 1% GlutaMAX 100×, 1% NEAA 100×, 0.008% (v/v) beta-mercaptoethanol, and mouse LIF] in the presence of ascorbic acid (50 μg/ml final). All cells were negative for mycoplasma infection. Cell lines were authenticated by gene expression analysis.

### Cell culture for 10X scRNA-seq

STEMCCA reprogramming was carried out as previously reported [43]. Briefly, STEMCCA MEFs [43, 99] were thawed in mESC media, on the next day cells were split into a 12-well plate (10 k cells per well) and cultured in mESC media with 15% FBS and AA, the following day the reprogramming was induced by adding doxycycline (2 μg/ml final). Medium was refreshed every 2 days with doxycycline and ascorbic acid (50 μg/ml final).

### Reprogramming experiments

Reprogramming experiments, unless stated otherwise, were performed by conditional induction of lentivirally delivered reprogramming factors. First, passage 1 MEFs at around 70% confluency were transduced with concentrated lentiviral supernatants. Lentiviruses were generated using HEK cells separately for two constructs: tetO-FUW-OSKM, Addgene cat. 20321 [100] and FUW-M2rtTA, Addgene cat. 20342 [101] with the calcium precipitation method. Supernatants with lentiviral particles were concentrated using lenti-X-concentrator (Takara, 631231) in 1:100 ratios. Infection with a pool of equal volumes of both constructs was carried out overnight, followed by 12 h culture in MEF medium and 1:6 split. Cells were then sorted using FACS (described below) in order to isolate homogeneous population with regard to allelic inactivation of the X-GFP transgene (either Xi-GFP or Xa-GFP), as described [102]. For the isolation of day 0 time point of single-cell transcriptome analysis, 96 Xi-GFP cells were sorted into a 96-well multiwell plate with 2 μl lysis buffer, followed by initial the steps of the scRNa-seq Smart-seq2 protocol (see below). For the isolation of day 0 time point of ATAC-seq analysis, 20,000 cells were sorted into a 1.5-ml centrifuge tube, followed by initial steps of Omni-ATAC-seq protocol (see below). Remaining cells were plated for reprogramming directly after sorting, 50,000 cells per one well of a 12-well plate. Reprogramming was induced by doxycycline (2 μg/ml final) in mESC medium in the presence of ascorbic acid (50 μg/ml final). The medium together with doxycycline and ascorbic acid was replaced every 2 days throughout reprogramming experiments. iPSC controls were derived using the same reprogramming system by picking ESC-like colonies at day 14 of reprogramming and subsequent culture for four passages without dox and ascorbic acid.

### Fluorescent activated cell sorting

For cell sorting, cells were dissociated using trypsin (0.25%) digestion. For X-GFP+/− cell sorting, dissociation was followed by washing in the incubation buffer (1× PBS, 0.5% BSA, 2 mM EDTA) and filtered through a Falcon® 40 μm Cell Strainer (Corning, cat. 352340). For sorting SSEA1+ cells, dissociation was followed by a wash in incubation buffer and 40 min of incubation with primary antibody anti-SSEA1 coupled with PE (Mouse IgM, R&D, FAB2155P, Clone MC-480, conc. 1 μl SSEA1-PE Ab / 5 × 10^6^ cells). Stained cells were subsequently washed in the incubation buffer to remove residual unbound antibody and passed through a cell strainer. Cell death exclusion was always applied by staining with DAPI (Sigma-Aldrich cat. D9542-50MG). Sorting was performed on a BD FACS Aria III or BD Influx (BD Biosciences) and performed by operators at the KU Leuven FACS core.

### Single-cell RNA sequencing (scRNA-seq)

Briefly, SSEA1+ reprogramming intermediates were sorted into single wells, each with 2 μl of the lysis buffer [0.2% Triton X100, 1:20 RRI (Recombinant RNase Inhibitor, Takara, 2313A)] and snap-frozen in − 80 °C until cells from all time points were collected. Next, all samples were processed following the Smart-seq2 protocol [103]. cDNA synthesis was done starting from 2.3 μl of input RNA, followed by library preparation from 1/5 dilution of cDNA using the Nextera XT kit (Illumina, FC-131-1096). Indexing was performed with the Nextera XT index Kit V2 (Illumina, FC-131-2003). The quality of cDNA and individual libraries was assessed using an Agilent 2100 Bioanalyzer system. Libraries were sequenced on an Illumina Nextseq 500 using single-end 50 bp mode yielding on average 0.5 million reads.

For Fig. 4D and S4E-G, cells were FACS sorted into 2.5 μl GpC methylase reaction mix, comprising 1× M.CviPI reaction buffer, 2 U M.CviPI (Bioké, M0227S), 160 μM S-adenosylmethionine (SAM), 1 U/μl RNAsein (Thermo Fisher, AM2694), and 0.1% IGEPAL (Sigma-Aldrich, CA-630). The samples were incubated at 37 °C for 15 min to label open chromatin after which 5 μl RLT plus buffer was added. The mRNA was captured using oligo-dT-coated paramagnetic beads on an automated liquid-handling robotics platform (Hamilton), following the G&T-seq procedure [104] with additional wash step. The beads were resuspended in 5 μl reverse transcription mix; 4 mM dNTPs, 1 μM TSO (IDT), 6 mM MgCl2, 1 M Betaine, 1× superscript II First-strand buffer, 5 mM DTT, 2.5 U RNase inhibitor (Thermo Fisher, R0192), and 50 U superscript II reverse transcriptase (Thermo Fisher, 18064022) and incubated at 42 °C for 2 min with mixing at 2000 rpm, 42 °C for 60 min and 1500 rpm, 50 °C for 30 min, and 1500 rpm and 60 °C for 10 min and 1500 rpm. cDNA amplification was performed by adding 7.5 μl cDNA amplification master mix, comprising 1× KAPA HiFi HotStart ReadyMix (KAPA biosystems, KR0370) and 0.1 μM IS PCR primer (IDT), followed by incubation at: 98 °C for 3 min, 23 cycles of 98 °C for 20 s, 67 °C for 15 s, 72 °C for 6 min, and finally, 72 °C for 5 min. The amplified cDNA was purified in a 1:1 ratio using AMPure beads (BeckmanCoulter, A63880)) and eluted in 25 μl nuclease-free water. Library preparation was performed using the Nextera XT kit (Illumina, FC-131-1096) according to the manufacturer’s instructions using 1:4th volumes starting from 250 pg cDNA. Final libraries were 96-plex pooled and SPRI purified using 0.66:1 ratio and quantified using qPCR while fragment size was assessed on the bioanalyzer. Libraries were 192-plex sequenced on an Illumina HiSeq 4000 platform in 50 bp single-end mode.

### 10X library preparation

To obtain single-cell suspensions, cells at day 16 of reprogramming were washed twice with PBS without Ca/Mg, detached using 0.25% Trypsin incubated at 37 °C for 5 min; cells were resuspended in 2 ml 0.04% BSA/PBS at the concentration of 1 million cells/ml.

Single-cell suspensions were converted to barcoded scRNA-seq libraries by using the Chromium Single Cell 3’ Library, Gel Bead & Multiplex Kit, and Chip Kit (10X Genomics). Samples were processed using kits pertaining to Chromium V2 chemistry of 10X genomics. Libraries were sequenced using HiSeq 4000 in paired-end mode.

### Assay for transposase-accessible chromatin using sequencing (ATAC-seq)

ATAC followed by sequencing was performed using the Omni-ATAC protocol [105]. Briefly, 20,000 sorted day 0 (Xi-GFP), SSEA1+/Xi-GFP day 8, 9, 10, and 12 reprogramming intermediates, and iPSC were pelleted at 500 RCF at 4 °C for 5 min in a fixed-angle centrifuge, and then the cells were gently washed once with 50 μl of cold PBS. Next, the cell pellets were resuspended in 50 μl of ATAC-lysis buffer (10 mM Tris HCl pH 7.4, 10 mM NaCl, 3 mM MgCl2, 0.1% Tween-20, 0.1% NP40, and 0.01% Digitonin) and incubated on ice for 3 min. Wash out lysis with 1 ml of cold ATAC-lysis buffer containing 0.1% Tween-20 but No NP40 or digitonin and invert tube 3 times to mix. Nuclei were pelleted at 500 RCF for 10 min at 4 °C in a fixed-angle centrifuge. After discarding all supernatant, nuclei were resuspended in 50 μl of transposition mixture (25 μl 2× TD buffer, 2.5 μl transposase (100 nM final), 16.5 μl PBS, 0.5 μl 1% digitonin, 0.5 μl 10% Tween-20, and 5 μl H2O) (Nextera DNA Sample Preparation Kit, Illumina, FC-121-1030). The reaction was performed at 37 °C for 30 min in a thermomixer with 1000 RPM mixing. The transposed DNA was purified using a Zymo DNA Clean and Concentrator-5 Kit (D4014). DNA libraries were PCR amplified using NEBNext HighFidelity 2× PCR Master Mix (Bioke, M0541), and size selected for 200 to 800 bp using homemade Serapure beads [106]. Library concentrations were quantified using Qubit dsDNA HS (High Sensitivity) Assay Kit (Invitrogen, Q32854) and equimolar amounts were pooled for paired-end sequencing on an Illumina NextSeq 500 instrument (Illumina) to yield ~ 100 million, 75-bp-long reads per sample.

### Smarts-seq2 scRNA-seq read processing

Reads were mapped to the mouse reference genome (mm10, GRCm38.p5) using STAR 2.5.3a [107] and GENCODE vM16 annotation file. The alignment was performed with default parameters and --sjdbOverhang set to 74 and output to sorted BAM files. Uniquely aligned reads were quantified using the featureCounts function from the R Bioconductor package “Rsubread” (version 1.5.2) [108].

### 10X scRNA-seq library preparation and read processing

Raw read counts were generated using CellRanger (2.1.1) and reference genome and annotation mm10 (1.2.0). Raw read count matrix was imported to Seurat R package [109] v3.1.2. Only cells that contained more than 1500, less than 6000 expressed genes, and less than 9% of mitochondrial RNA were retained. Subsequently, reads were normalized using scale factor and log-transformed.

### Smart-seq2 scRNA-seq allele-specific read processing

For allele resolution analyses, reads were processed as previously described [47]. Briefly, reads were mapped to the same reference genome release in which SNP positions were substituted by N base (referred to as N-masked mm10). N-masking was performed with the SNPsplit software (Version 0.3.2 released (29-03-2017)) [110] supplied with the list of strain-specific SNPs (129S1_SvImJ and CAST_EiJ) from the Sanger Mouse Genomes project database (mgp.v5.merged.snps_all.dbSNP142.vcf.gz). N-masking was done in dual hybrid mode and resulted in the identification throughout the entire genome of 20,563,466 SNP positions unique for either strain, of which 634,730 on the X Chromosome. Next, reads were aligned to the N-masked mm10 genome using STAR 2.5.3a with parameters disabling soft-clipping of incompletely aligned reads (--alignEndsType EndToEnd --outSAMattributes NH HI NM MD). Reads aligned to the N-masked reference genome were then split into two BAM files containing only strain-specific reads (on average ~ 5% of total mapped reads for either allele) using SNPsplit (version 0.3.4). Unclassified reads were not used for subsequent allele-specific analysis. Using the allelic BAM files, variant calling files (VCF) were generated using samtools’ mpilup command (version 1.7) [111], and merged using bcftools (version 1.5) [112]. An allelic count matrix was created by retaining counts from SNP positions and annotating these positions with their respective genes, where only genes covered by 4 SNPs or more are kept. Allelic data was size factor normalized by dividing allele-specific gene counts by total counts of that cell, and this was then multiplied by 10,000 and log-transformed.

Allele-specific X/A ratios were calculated by retaining expressed genes (the sum of allelic reads for each gene in each cells > 0) and dividing the median normalized expression of either X-allele by that of both autosomes.

For classification of X-linked genes, total and X*-Cast* expression was counted as downregulated or upregulated when it decreases or increases by 25%, respectively. *Mus*/*Cast* are biasedly expressed towards either allele when there is a minimum of 25% expression difference.

### scRNA-seq clustering and gene expression analysis

scRNA-seq data analysis was performed using the Seurat R package [113] (https://satijalab.org/seurat/, version 3.1.1). Cells were retained for analysis if they passed quality criteria: detected genes > 4000 and < 10,000, and percentage mitochondrial RNA < 8%. Read counts were normalized using standard Seurat normalization: for each cell, the gene counts were divided by total counts and multiplied by a “scaling factor” of 1 × 10^5^, followed by log transformation tSNE dimensionality reduction, performed using the runTSNE function with parameters: dims = 1:15, seed.use = 1234. Graph-based clustering was performed using FindNeighbors (dims = 1:15) and FindClusters (resolution = 0.5) functions. Pseudotime trajectory was inferred using Monocle v2 [114], following the ordering workflow described (http://cole-trapnell-lab.github.io/monocle-release/docs/#constructing-single-cell-trajectories). Briefly, genes that define progress through reprogramming were identified using the differentialGeneTest() function. Next, dimensionality reduction was performed using the reduceDimension (max_components = 2, method = “DDRTree”) function. Finally, cells were ordered using the orderCells() function.

### Gene expression integration

For integration with published datasets and 10X data generated in this study a published scRNA-seq reprogramming atlas was downloaded from GSE115943. Filtered and normalized gene expression data for 251,203 cells were subsampled randomly to 50,000 cells with set.seed (1234). Integration of [59] dataset, smart-seq2 data and 10X data was performed using Seurat’s canonical correlation analysis (CCA) integration tool. Anchors for integration were found using FindIntegrationAnchors function with dims.used: 40, k.filter: NA, k.anchors: 30, k.score: 30 parameters and data was integrated across all features. Integration-based UMAP was constructed using runUMAP function with dims.used: 40.

### Transcriptional burst analysis

Transcriptional burst analysis was performed as in [22, 67]. Briefly, we normalized raw allelic counts using RPKM (reads per kilobase million) method. To differentiate missing data (NaN) from not expressed genes, we annotated missing allelic data (genes with expression but no allelic reads) to NaN and genes without expression to 0. Next, we estimated parameters of bursting kinetics with the publicly available function *txburstML.py* from (https://github.com/sandberg-lab/txburst) [67]. This function infers transcriptional burst parameters with the two-state model of transcription, as described in the “Results” section, toestimate burst frequency (*k*_on_) and burst size (*k*_syn_/*k*_off_) [22, 67]. Plots were performed with seaborn (v0.10.0) [115]. Wilcoxon rank-sum statistic was conducted for significant testing with scipy (v1.2.1) with the function scipy.stats.ranksums.

### AUCell signature enrichment analysis

AUCell (1.8.0) [77] was used for the quantification of gene set signatures in each cell. The enrichment of the gene sets was calculated as an area under the recovery curve (AUC) across the ranking of all genes in a particular cell, whereby genes are ranked by their expression value. Next, AUC is used to calculate whether a critical subset of the input gene set is enriched at the top of the ranking for each cell. Unless stated otherwise, the AUC threshold was defined automatically based on the AUC score distribution across cells using mixtools package [116] (1.2.0). Gene sets were defined and published in [59].

### Gene regulatory network inference

Gene regulatory networks were inferred using pySCENIC (0.9.15; python implementation of SCENIC) [77, 78] in Python (3.6.9) normalized counts were used to generate co-expression modules using GRNboost2 algorithm [117] implemented in arboreto package (v0.1.3). Next, GRNs were inferred using pySCENIC (with default parameters and mm10__refseq-r80__10kb_up_and_down_tss.mc9nr and mm10__refseqr80__500bp_up_and_100bp_down_tss.mc9nr motif collections) resulting in the matrix of AUCell values that represents the activity of each regulon in each cell. To control for stochasticity, a consensus GRN was generated by merging results from five independent repeated pySCENIC runs. If regulons were identified in multiple pySCENIC runs, only the regulon with the highest AUC value was retained. Regulon-based UMAPs were generated using the runUMAP (dims = 1:15, seed.use = 1234) function in the Seurat package. Cluster-specific regulons were identified using FindAllMarkers (only.pos = TRUE, logfc.threshold = log(1)) function in Seurat package.

### Gene regulatory network visualization

In order to generate a visualization of the GRN, first additional filtering steps were performed. TF-target connections from 5 pySCENIC runs were filtered to retain only connections that appeared in all 5 runs and those with connections weight > 1. To remove regulons active in a small number of cells, only regulons active in at least 10 cells were kept. The network was generated using tidy graph package (v1.1.2, https://github.com/thomasp85/ggraph). Centrality degree was calculated, and only nodes with centrality > 50 are labelled. Nodes were colored using scaled expression data of the gene corresponding to given TF or target in the network. To represent the activity of the network in each of the defined states, scaled expression data were averaged across all cells from the given state. The network was plotted using ggraph package (v2.0.0, https://github.com/thomasp85/ggraph) with a size defined by the centrality degree and “layout” parameter set to “stress.”

### Integration of regulon data

Regulon data from SCENIC GRN inference in [59] dataset with *Mus* background, smart-seq2 data from this study and 10X data from this study, were integrated using Seurat’s canonical correlation analysis (CCA) integration tool. Anchors for integration were found using FindIntegrationAnchors function with dims.used = 20, k.filter = NA, k.anchors = 30, and k.score = 30 parameters, and data was integrated across all features. Integration-based UMAP was constructed using runUMAP function with dims.used: 20.

### ATAC-seq alignment and peak calling

Paired-end ATAC-seq raw data were analyzed using the ENCODE ATAC-seq pipeline (v1.1.5) with default parameters as described previously (https://github.com/ENCODE-DCC/atac-seq-pipeline). Reads were aligned to the ENCODE mouse reference genome GRCm38/mm10 (ENCSR425FOI). Alignment and peak calling results were integrated using the DiffBind (v3.8) resulting in read count matrix which was subsequently normalized by size factor and log2 transformed using the DESeq2 [118] (v.1.21.22).

### Allele resolution ATAC-seq

For allele resolution analyses, the ENCODE ATAC-seq pipeline was adapted to accommodate the allele-specific splitting of sequencing reads. First, N-masked reference genome was used (see above) for alignment. Mapping step was adjusted by removing --local parameter to enable end-to-end alignment. Second, mapped reads after removing duplicates, low-quality reads and mitochondrial regions were used as input for SNPsplit together with the list of strain-specific SNPs (129S1_SvImJ and CAST_EiJ) from the Sanger Mouse Genomes project database (mgp.v5.merged.snps_all.dbSNP142.vcf.gz) and split into two BAM files containing only strain-specific reads. The resulting, strain-specific BAM files were then used as input for the peak calling steps in the ENCODE ATAC-seq pipeline. Alignment and peak calling results were integrated using DiffBind (v3.8) resulting in allelic read count matrices. Genomic tracks were generated using bamCoverage function from deepTools (3.3.1) with default parameters and binsize set to 1. For Figs. 1 and 2, at each time point, only regions where the sum of reads from both alleles was higher or equal to 10 were retained, which was important to detected enhanced chromatin accessibility. Allelic reads were normalized by dividing the reads from each allele of the sample by the number of total reads in the sample and multiplied by 10,000 and log1p transformed. For the calculation of allelic chromatin accessibility ratios in Fig. 5, regions with at least 9 counts as the sum of both alleles at every time point were retained (total of 750 regions). Allelic ratios were calculated as the ratio of *Mus* to total: (*Mus*/(*Mus* + *Cast*). A ratio from 0 to 0.15 means X-*Cast* mono-accessibility; a ratio from 0.15 to 0.85 is defined as biallelic accessibility; a ratio from 0.85 to 1 means X*-Mus* mono-accessibility.

### ChromHMM

ChromHMM (v.1.18) [75] was used to annotate chromatin states. First, the aligned reads for selected chromatin marks in ESCs and MEFs were binarized with ChromHMM’s BinarizeBam command. Next, the chromatin-state model was constructed with ChromHMM’s LearnModel command. Resulting chromatin states were interpreted based on the output features and literature (Additional file 3: Table S2) [54, 75, 76]. Next, ATAC-seq reprogramming data was annotated using the defined ChromHMM states using bedtools (v2.28.0) with the function intersectBed. Only the region assigned to the chromatin state with the highest degree of overlap was preserved. For Fig. 5, enhancers and promoters were defined by merging all chromatin states that include “enhancer” or “promoter.” ATAC-seq regions were annotated as explained above. The average estimated time for X-linked enhancers and promoters to become accessible during reprogramming was inferred using the log-linked Gaussian generalized linear model to relate the ATAC-seq allelic ratios to reprogramming time points.

### ATAC-seq clustering analysis

Top 65,535 most variable non-allelic ATAC-seq normalized regions (out of 223,596 total regions) among the different reprogramming time points were clustered with the *k-*means function from the stats package (v3.6.1) with 10 centers. The same analysis was performed for the 5357 X-linked non-allelic ATAC-seq normalized counts and for clustering allelic accessibility ratio data across the reprogramming time points.

To assess the distance to the nearest day 0 biallelically accessible regions and enrichment of TFs, enrichment values were calculated using bedops (v2.4.36) with the function bedmap [119]. Statistical significance of differences between enrichment levels in different clusters was measured using the Wilcoxon rank test.

### Motif enrichment analysis

Motif enrichment analysis in ATAC-seq biallelic regions during reprogramming and enhancer and promoter regions, compared to over 50,000 random background genome regions, was performed using findMotifsGenome.pl tool from HOMER software (v4.11.1). The option -size was set to − 250,250, the number of motifs to optimize was set to − S = 15 and motif length (− len) to 6, 8, 10, 12, and 16. For Fig. 5, enriched motifs were filtered for motifs with *p* value < 0.05.

### ChIP sequencing (ChIP-seq) analysis

For the analyses of TF and histone mark enrichment, published ChIP-seq data for pluripotency factors and chromatin marks in ESCs and MEFs were reanalyzed ([76]: GSE90893 [120];: GSE25409 and [121]: GSE36905). ChIP-seq data were analyzed using the ChIP-seq pipeline from the Kundaje laboratory (version 0.3.0; https://github.com/kundajelab/atac_dnase_pipelines). The enrichment values were calculated by summing the score within accessible regions using bedops (2.4.36) [119].

### Genomic regions annotations

*Cis*-regulatory regions were annotated using GREAT (v4.0.4) [122] using mouse NCBI build 38 (UCSC mm10, Dec/2011) as species assembly with single nearest gene of 1000 kb as association rule.

### Data visualization

Unless stated otherwise, analysis was conducted in R v3.6.1 (2019-07-05) and figures were produced using the R packages ggplot2 v3.1.1, pheatmap v1.0.12 and gplots v 3.0.1.1. All boxplots represent the median of the data and the lower and upper hinges correspond to the first and third quartiles (the 25th and 75th percentiles).

## Supplementary Information

**Additional file 1: Fig. S1-S6.** Supplementary figure legends and supplementary figures (Fig. S1-S6).

**Additional file 2: Table S1.** Allelic normalized accessibility of XX (CM2) and XY (CM7) mESCs on the X chromosome. Regions are ordered from largest to smallest differentially accessible region on the *Mus* allele from XY mESCs compared to *Mus* allele from XX mESCs.

**Additional file 3: Table S2.** ChromHMM emission probabilities. Each row represents a distinct chromatin state based on the putative annotation. Cells show the modelled frequency of each histone mark in either ESCs or MEFs.

**Additional file 4: Table S3.** Wilcoxon rank-sum test p.values of median distance to nearest day 0 biallelically accessible region (Additional file 1: Fig. S5D).

**Additional file 5: Table S4.** List of regulons with TFs and their targets as defined by SCENIC in Fig. 6.

**Additional file 6: Table S5.** Numbers of Regulon targets per chromosome for all active Regulons during reprogramming.

**Additional file 7.** Review history

## Data Availability

All raw and processed sequencing data generated in this study are available in the NCBI Gene Expression Omnibus under GEO SuperSeries with accession number GSE153847 (https://www.ncbi.nlm.nih.gov/geo/query/acc.cgi?acc = GSE153847) [123]. All codes are available on the Pasque Lab Github account (https://github.com/pasquelab/X_dosage_GenomeBiology_2021) [124] and Zenodo (10.5281/zenodo.5541251) [125] licensed under the MIT License.
