# Supplementary material for: Enhanced chromatin accessibility contributes to X chromosome dosage compensation in mammals
Source: Genome Biol. 2021 Nov 1;22:302. doi: 10.1186/s13059-021-02518-5 (PMC8558763; doi:10.1186/s13059-021-02518-5)
Supplement: Supplementary file 1 — Additional file 1: Fig. S1-S6. Supplementary figure legends and supplementary figures (Fig. S1-S6). [file 13059_2021_2518_MOESM1_ESM.pdf]

Additional file 1: Figures S1-S6.

FIGURE LEGENDS

Fig S1. Chromatin hyperaccessibility on the Xa in somatic female cells.

A. ATAC-seq tracks for allelic chromatin accessibility at promoter regions of representative X-linked gene regions. *Cast* regions are represented in red whereas *Mus* regions are represented in blue.

B. Violin plot combined with boxplot showing chromosome to autosome accessibility ratio for the *Mus* allele. The dashed line indicates the expected chr/A chromatin accessibility ratio when the indicated chromosome has the same level of accessibility as an autosome.

C. Violin plot combined with boxplot showing chromosome to autosome chromatin accessibility ratio for the *Cast* allele. The dashed line indicates the expected chr/A chromatin accessibility when the indicated chromosome has the same level of accessibility as an autosome.

Fig S2. Chromatin accessibility changes during MEFs reprogramming to pluripotency.

A. Principal component analysis (PCA) of all accessible regions from different stages of reprogramming. Each colored dot represents a sample isolated at a different time point.

B. Heatmap showing k-means clustering of non-allelic autosomal chromatin accessibility with the top 65535 most variable regions across the time course of reprogramming (log2-normalized read counts).

C. Bar plot with total number of peaks per time point. Accessible peaks were defined with a threshold of > 50 counts.

D. Bar plot with number of peaks on the X chromosome per time point. Accessible peaks were defined with a threshold of > 50 counts.

E. ATAC-seq signals for non-allelic chromatin accessibility of pluripotency markers and somatic markers.

F. Violin plot combined with boxplot showing chromosome to autosome accessibility ratio for the Cast allele in iPSCs. The dotted line indicates the expected chr/A chromatin accessibility when the indicated chromosome has equal accessibility as an autosome.

G. Violin plot combined with boxplot showing X chromosome to autosome accessibility ratio for the *Cast* allele during reprogramming. The dashed line indicates the expected X/A chromatin accessibility ration when the X chromosome has equal accessibility as an autosome. A Wilcoxon rank-sum test was used for significance testing.

H. Violin plot combined with boxplot showing normalized accessibility of autosomal regions in MEFs and iPSCs for the *Cast* and the *Mus* allele. Wilcoxon rank-sum test used for significance testing. Reanalysis of data from [56].

I. Dot plot with X chromosome to autosome accessibility ratio (fold change) for *Mus* allele (blue) and *Cast* (red) in XX and XY mESC lines. Reanalysis of data from [56].

J. Violin plot combined with boxplot showing normalized accessibility of the Xa chromosome and the median of all the autosomes for the *Mus* allele in two XY mESC lines (reanalysis from [56]). A Wilcoxon rank-sum test was used for significance testing.

K. ATAC-seq signals for allelic chromatin accessibility of *Klhl14* and *Prkx* transcript regions in mESCs. Allele of origin is indicated in blue for *Mus* allele and in red for *Cast*. Reanalysis of data from [56].

Fig S3. Single-cell transcriptome landscape of cell fate reprogramming to iPSCs.

A. Single cells ordered by pseudotime trajectory (left) and for cells that were isolated at specific time points (right). Each dot represents a single cell colored by their isolation time point.

B. Bar plot showing the proportion of single cells that fall in each cluster (C0-C5). Colors indicate the isolation time point.

C. Schematic representation of scRNA-seq datasets used in this study.

D. UMAP of integrated single-cell gene expression colored by reprogramming isolation time point. Each dot represents a cell.

E. UMAP of single-cell gene expression colored by gene signature. Each dot represents a cell and colors indicate the gene signature.

F. Density plots of gene signatures found in the integrated dataset. Gene signature enrichment analysis was performed with AUCell. Red vertical line indicates a binary signature activity threshold set automatically by AUCell. Dashed black line indicates manually adjusted signature activity threshold.

G. Bar plot showing the proportion of single Smart-seq2 scRNA-seq cells from this study expressing each gene signature. Colors indicate the isolation time point.

H. tSNE visualization of gene expression levels from single Smart-seq2 cells from this study. Each dot represents a cell and colors indicate the different gene signature.

I. Box plot with the *Mus*/*Cast* allele ratio of X-linked genes between the clusters ordered by XCR timing. The colors represent the XCR category as defined in [47] (light blue for early genes, dark blue for intermediate, light green for late, dark green for very late and pink for escapees). Ratios were calculated by dividing maternal by total reads (*Mus*/*Mus*+*Cast*), a ratio < 0.15 indicates *Cast* expression and a ratio > 0.85, *Mus* expression.

J. Normalized expression levels of early reactivating genes on the Xi compared to total expression of *Xist* plotted along the reprogramming pseudotime trajectory.

Fig S4. XCU erasure during reprogramming.

A. Median expression of autosomal genes in cells from cluster C0 and C5. Wilcoxon rank-sum test used for significance testing.

B. Median expression of genes from X-*Cast* (red), X-*Mus* (blue) alleles and autosomal (green) genes from both alleles shown along the reprogramming pseudotime (left) and in Clusters C0 and C5 (right)

C. Box plot representing the number of genes grouped according to two variables. Top: genes classified based on expression change in iPSCs relative to MEFs (eg. ‘Higher’ contains genes for which total expression from both alleles increases in iPSCs relative to MEFs after reprogramming). Bottom: genes are further subgrouped depending on the change in the expression of the Cast allele of a gene in iPSCs relative to MEFs (eg ‘Lower’ means that gene expression on Cast allele decreased after reprogramming).

D. Box plots with burst frequency and burst size for all chromosomes in C0-C5 cluster cells in the *Cast* allele.

E. iPSCs grouped by the X-linked genes mean allelic expression ratio. Bi-allelic expression: 0.15 < mean ratio < 0.85, *Cast* monoallelic expression: mean ratio < 0.15 and *Mus*: > 0.85.

F. Density plot with normalized expression of X-*Mus* genes from XX iPSC (dark grey), X-*Cast* genes from XX iPSC (light grey), and X-*Cast* genes from X-*Cast* O-*Mu*s iPSC (red) on the top plot. On the right plot, X-*Mus* genes from XX iPSC (dark grey), X-*Cast* genes from XX iPSC (light grey) and X-*Mus* genes from X-*Cast* O-*Mu*s iPSC (blue). Wilcoxon rank-sum test used for significance testing.

G. Distribution of burst frequency and burst size of autosomal and X-linked genes on the *Cast* allele in X-*Cast* O-*Mus*, X-*Mus* O-*Cast* and X-*Cast* X-*Mus* iPSCs. A Wilcoxon rank-sum test was used for significance testing.

Fig S5. Allele-resolution chromatin accessibility profiling of the inactive X chromosome gaining accessibility during reprogramming to iPSCs.

A. Mean accessibility ratio of the X, 2 and 8 chromosomes to autosomes (log2-normalized read counts) during reprogramming.

B. Heatmap showing K-means clustering of allelic X-linked chromatin accessibility regions during reprogramming (log2-normalized read counts). The colored bar refers to the different clusters.

C. Heatmap with allelic ratio (*Mus*/(*Mus*+*Cast*)) of all informative X-linked regions during reprogramming.

D. Box plot with the median distance to nearest day 0 biallelically accessible region (Mb) for each X-linked chromatin accessibility cluster during reprogramming. Wilcoxon rank-sum test between clusters was performed to test significance. The significant p.values are indicated

with asterisks above the box plots: (∗∗∗) p.value = 0.0001–0.001; (∗) p.value = 0.01–0.05 = significant; p.value ≤ 0.05 (not significant).

E. Table with the association of X accessibility regions clusters with the closest genes.

F. Comparison of gene association of chromatin accessibility dynamics to that of transcription kinetics of XCR following iPSC reprogramming.

G. Enrichment of TF motifs in X-linked enhancers (top) and promoter (bottom) regions. Only enrichments with p.value ≤ 0.05 are shown. The color gradient represents the percentage of regions with enriched motif in the indicated group over 50000 random background genome regions. Enhancers and promoters were defined in mESCs and MEFs as described in Methods.

H. Violin plots indicating the sum score of enrichment levels of OCT4, SOX2, KLF4, c-MYC, ESRRB, PRDM14, NANOG and p300 occupancy for each accessibility cluster in ESCs. Wilcoxon rank-sum test was used to test significance. Stars indicate significance as in Figure legend S5D.

I. Genome browser tracks showing the genomic region belonging to Early 1 accessibility cluster at day 8 of reprogramming, for *Acot9* and *Sat1* X-linked genes.

Fig S6. GRN inference in integrated single-cell datasets.

A. Predicted GRNs for the somatic, intermediate and pluripotent cell states. Nodes represent TFs and edges point toward target genes. Nodes are colored by scaled normalized expression of genes within cells from each state indicated in the top right corner. Outlines highlight parts of the network with activity specific to each regulatory state. The size of the node is determined by the number of outgoing edges (target genes).

B. On the right, plots with average regulon activity of selected regulons in each cluster (C0-C5).

C. UMAPs of single-cell clustering based on regulon activity in integrated datasets. Each dot represents a cell. Colors indicate isolation time points (left) and study origin (right) (Smart-seq2 scRNA-seq cells from this study are colored in red, cells from [59] are colored in grey and 10X cells from this study in blue).

D. Heatmap with regulon activity of selected intermediate-specific regulons in cells expressing each signature (red = pluripotent, yellow = other, green = epithelial, turquoise = senescent, light blue = neural, purple = trophoblast and pink = MEF).

E. Number of ZFP42, HCFC1, TRP53 and TCF7L2 predicted targets on all chromosomes with SCENIC.

F. Percentage of X-linked genes to which ZFP42 is bound in mESCs. Data from [85].

G. X–Y plots showing the mean regulon activity (x-axis) and the percentage of TF motif enrichment (y-axis) for the overlapping TFs between GRN and motif enrichment analyses at

different time points during reprogramming. Each plot corresponds to a different time point (day 0, 8, 9, 10, 12 and iPSCs) and each dot corresponds to the values for a specific TF.

Fig S1

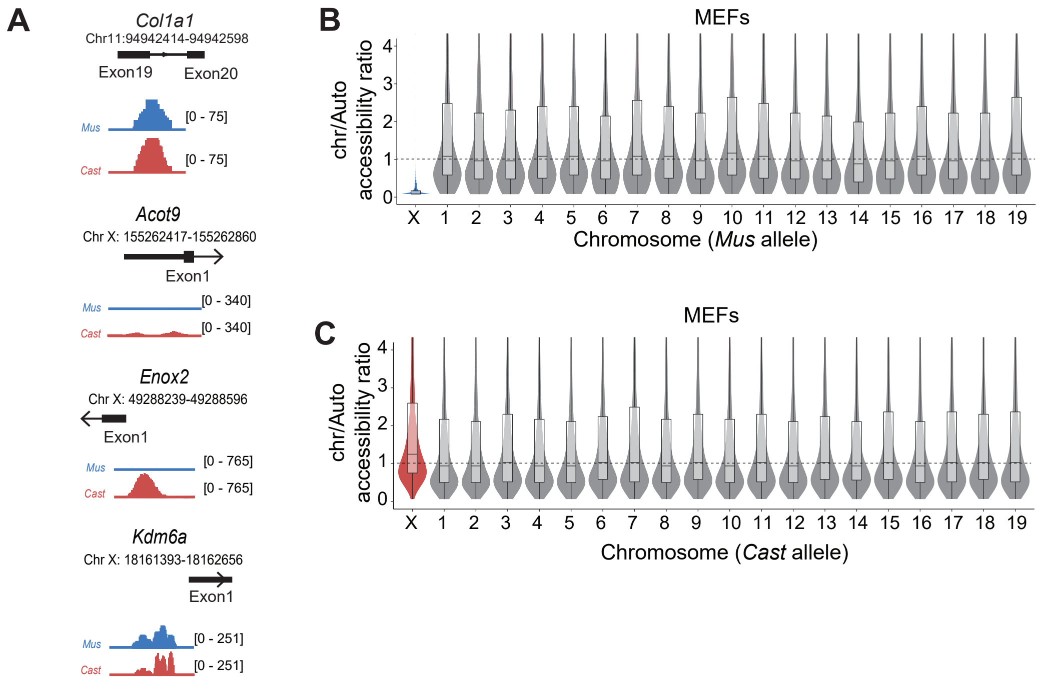
A
Col1a1
Chr11:94942414-94942598
Exon19
Exon20
Mus
[0 - 75]
Cast
[0 - 75]
Acot9
Chr X: 155262417-155262860
Exon1
Mus
[0 - 340]
Cast
[0 - 340]
Enox2
Chr X: 49288239-49288596
Exon1
Mus
[0 - 765]
Cast
[0 - 765]
Kdm6a
Chr X: 18161393-18162656
Exon1
Mus
[0 - 251]
Cast
[0 - 251]
B
MEFs
chr/Auto accessibility ratio
0 1 2 3 4
X 1 2 3 4 5 6 7 8 9 10 11 12 13 14 15 16 17 18 19
Chromosome (Mus allele)
C
MEFs
chr/Auto accessibility ratio
0 1 2 3 4
X 1 2 3 4 5 6 7 8 9 10 11 12 13 14 15 16 17 18 19
Chromosome (Cast allele)

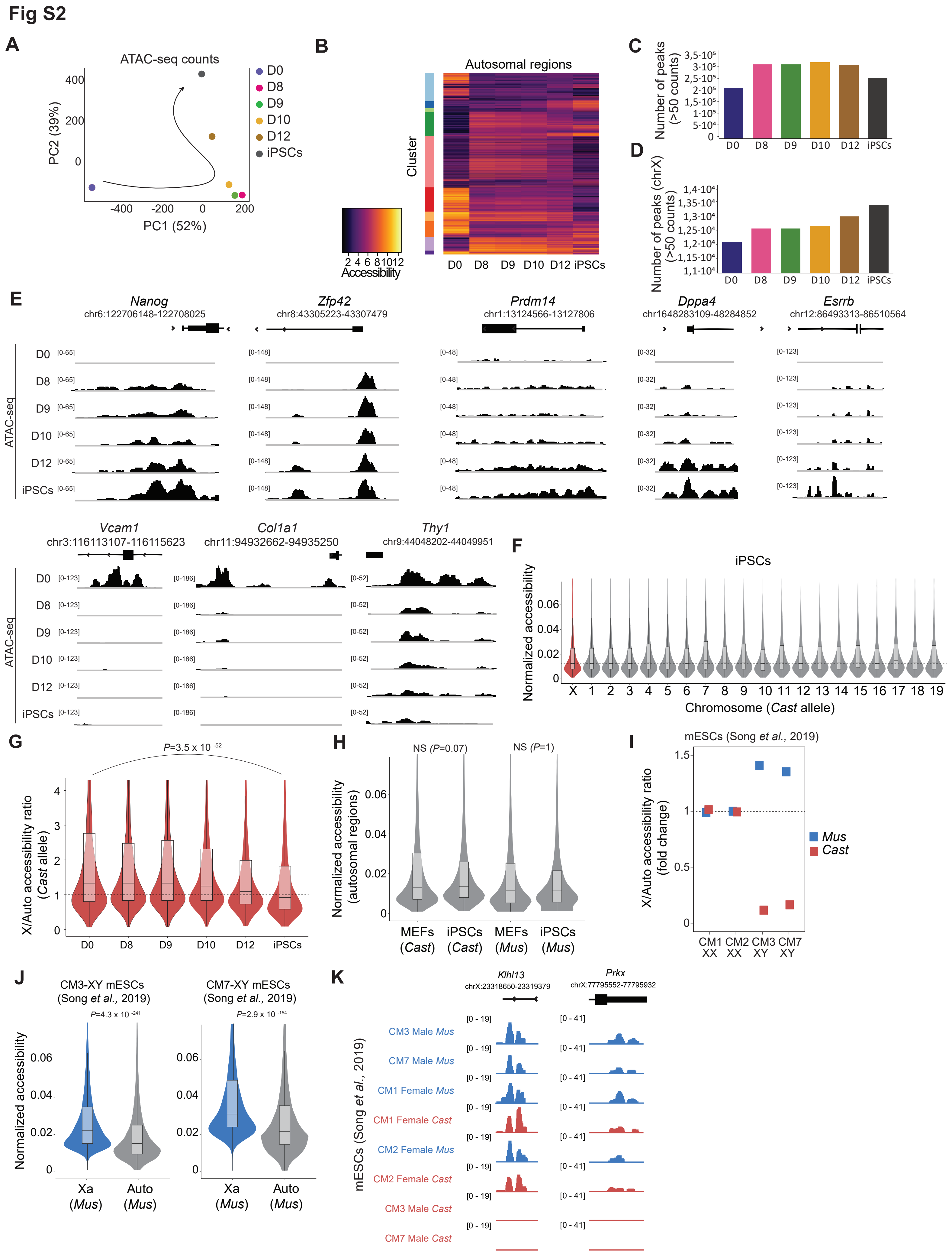

Fig S2
A
ATAC-seq counts
PC2 (39%)
PC1 (52%)
D0
D8
D9
D10
D12
iPSCs
B
Autosomal regions
Cluster
Accessibility
D0 D8 D9 D10 D12 iPSCs
C
Number of peaks (>50 counts)
D
Number of peaks (chrX) (>50 counts)
E
Nanog
chr6:122706148-122708025
Zfp42
chr8:43305223-43307479
Prdm14
chr1:13124566-13127806
Dppa4
chr1648283109-48284852
Esrrb
chr12:86493313-86510564
ATAC-seq
Vcam1
chr3:116113107-116115623
Col1a1
chr11:94932662-94935250
Thy1
chr9:44048202-44049951
F
iPSCs
Normalized accessibility
Chromosome (Cast allele)
G
P=3.5 x 10 -52
X/Auto accessibility ratio (Cast allele)
H
NS (P=0.07)
NS (P=1)
Normalized accessibility (autosomal regions)
MEFs (Cast) iPSCs (Cast) MEFs (Mus) iPSCs (Mus)
I
mESCs (Song et al., 2019)
X/Auto accessibility ratio (fold change)
Mus
Cast
CM1 XX CM2 XX CM3 XY CM7 XY
J
CM3-XY mESCs (Song et al., 2019)
CM7-XY mESCs (Song et al., 2019)
P=4.3 x 10 -241
P=2.9 x 10 -154
Normalized accessibility
Xa (Mus) Auto (Mus)
K
Klhl13
chrX:23318650-23319379
Prkx
chrX:77795552-77795932
mESCs (Song et al., 2019)
CM3 Male Mus
CM7 Male Mus
CM1 Female Mus
CM1 Female Cast
CM2 Female Mus
CM2 Female Cast
CM3 Male Cast
CM7 Male Cast

Fig S3

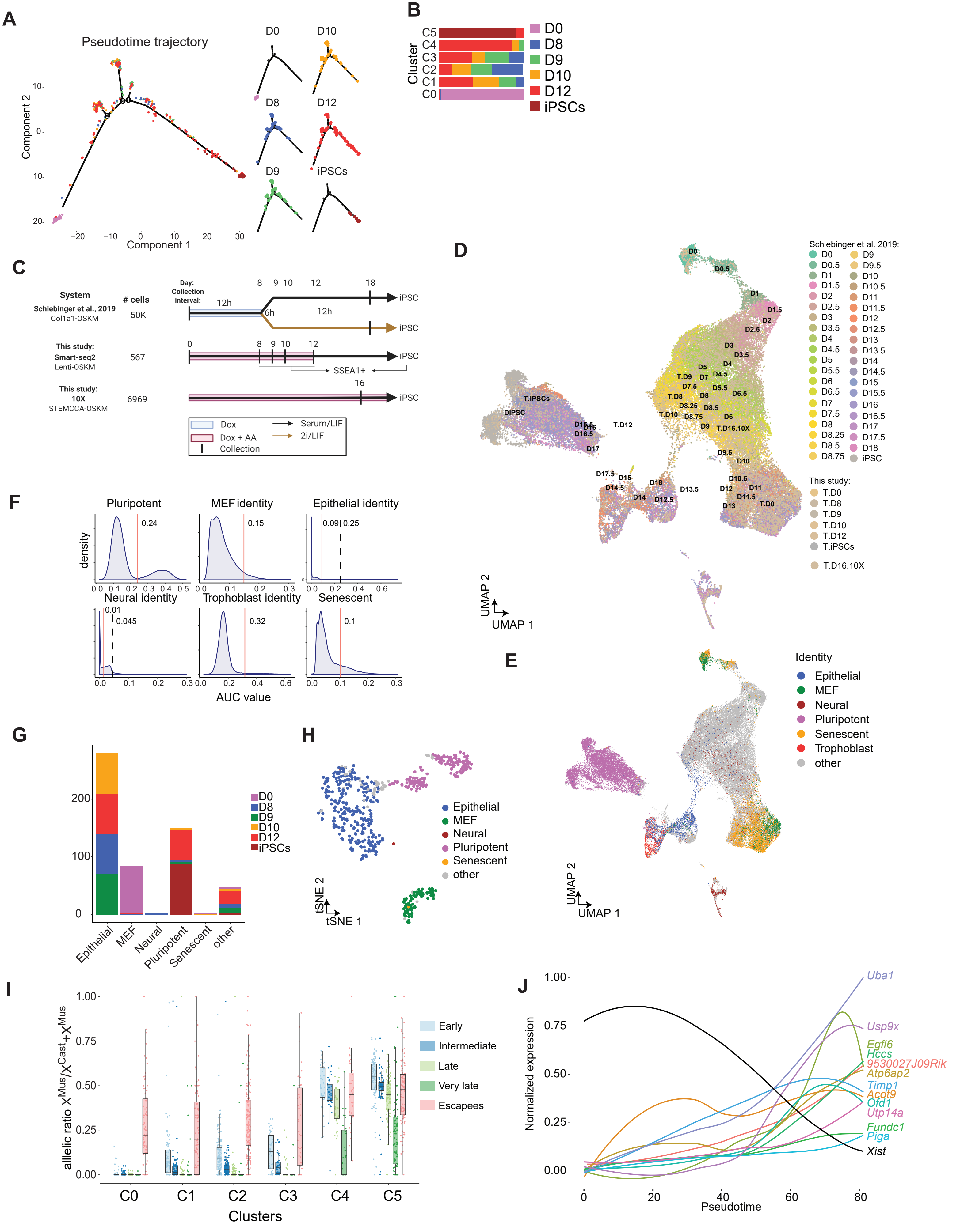

A
Pseudotime trajectory
Component 1
Component 2
D0
D10
D8
D12
D9
iPSCs
B
Cluster
C5
C4
C3
C2
C1
C0
D0
D8
D9
D10
D12
iPSCs
C
System
cells
Schiebinger et al., 2019
Col1a1-OSKM
50K
This study:
Smart-seq2
Lenti-OSKM
567
This study:
10X
STEMCCA-OSKM
6969
Day:
Collection interval:
12h
6h
12h
iPSC
SSEA1+
Dox
Dox + AA
Collection
Serum/LIF
2i/LIF
D
Schiebinger et al. 2019:
This study:
UMAP 2
UMAP 1
E
Identity
Epithelial
MEF
Neural
Pluripotent
Senescent
Trophoblast
other
F
Pluripotent
MEF identity
Epithelial identity
Neural identity
Trophoblast identity
Senescent
density
AUC value
G
Epithelial
MEF
Neural
Pluripotent
Senescent
other
H
tSNE 2
tSNE 1
I
Early
Intermediate
Late
Very late
Escapees
Clusters
J
Normalized expression
Pseudotime
Uba1
Usp9x
Egfl6
Hccs
9530027J09Rik
Atp6ap2
Timp1
Acot9
Ofd1
Utp14a
Fundc1
Piga
Xist

Fig S4

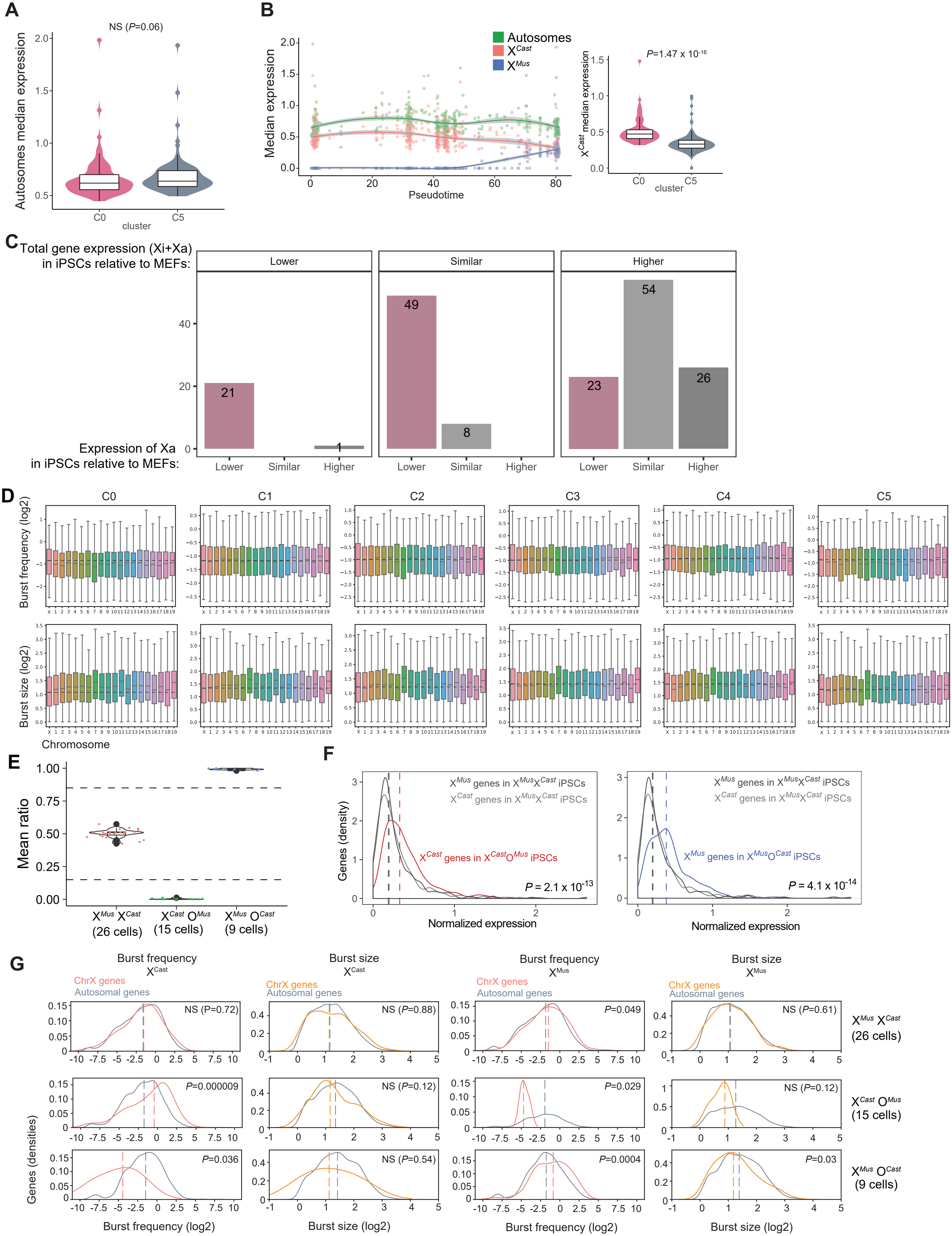
A
NS (P=0.06)
Autosomes median expression
cluster
C0
C5
B
Autosomes
X^Cast
X^Mus
Median expression
Pseudotime
P=1.47 x 10^-16
X^Cast median expression
cluster
C
Total gene expression (Xi+Xa) in iPSCs relative to MEFs:
Lower
Similar
Higher
Expression of Xa in iPSCs relative to MEFs:
D
C0
C1
C2
C3
C4
C5
Burst frequency (log2)
Burst size (log2)
Chromosome
E
Mean ratio
X^Mus X^Cast (26 cells)
X^Cast O^Mus (15 cells)
X^Mus O^Cast (9 cells)
F
X^Mus genes in X^Mus X^Cast iPSCs
X^Cast genes in X^Mus X^Cast iPSCs
X^Cast genes in X^Cast O^Mus iPSCs
X^Mus genes in X^Mus O^Cast iPSCs
P = 2.1 x 10^-13
P = 4.1 x 10^-14
Genes (density)
Normalized expression
G
Burst frequency
Burst size
ChrX genes
Autosomal genes
NS (P=0.72)
NS (P=0.88)
P=0.049
NS (P=0.61)
P=0.000009
NS (P=0.12)
P=0.029
NS (P=0.12)
P=0.036
NS (P=0.54)
P=0.0004
P=0.03
Genes (densities)
Burst frequency (log2)
Burst size (log2)

Fig S5

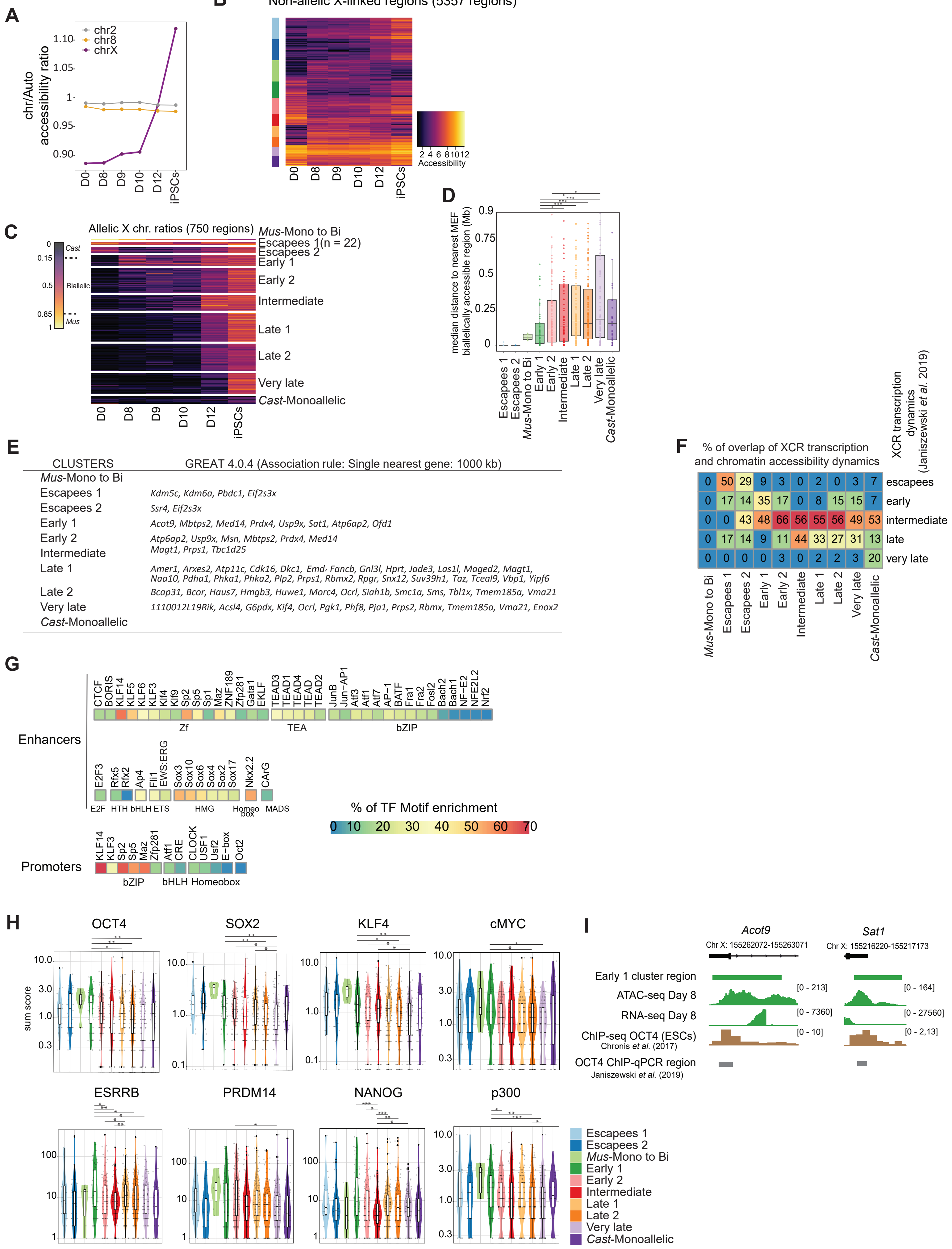

A
chr2
chr8
chrX
chr/Auto accessibility ratio
D0 D8 D9 D10 D12 iPSCs
B
Non-allelic X-linked regions (5357 regions)
Accessibility
C
Allelic X chr. ratios (750 regions)
Cast
Biallelic
Mus
Mus-Mono to Bi
Escapees 1(n = 22)
Escapees 2
Early 1
Early 2
Intermediate
Late 1
Late 2
Very late
Cast-Monoallelic
D
median distance to nearest MEF biallelically accessible region (Mb)
E
CLUSTERS
GREAT 4.0.4 (Association rule: Single nearest gene: 1000 kb)
Mus-Mono to Bi
Escapees 1 Kdm5c, Kdm6a, Pbdc1, Eif2s3x
Escapees 2 Ssr4, Eif2s3x
Early 1 Acot9, Mbtps2, Med14, Prdx4, Usp9x, Sat1, Atp6ap2, Ofd1
Early 2 Atp6ap2, Usp9x, Msn, Mbtps2, Prdx4, Med14
Intermediate Magt1, Prps1, Tbc1d25
Late 1 Amer1, Arxes2, Atp11c, Cdk16, Dkc1, Emd, Fancb, Gnl3l, Hprt, Jade3, Las1l, Maged2, Magt1, Naa10, Pdha1, Phka1, Phka2, Plp2, Prps1, Rbmx2, Rpgr, Snx12, Suv39h1, Taz, Tceal9, Vbp1, Yipf6
Late 2 Bcap31, Bcor, Haus7, Hmgb3, Huwe1, Morc4, Ocrl, Siah1b, Smc1a, Sms, Tbl1x, Tmem185a, Vma21
Very late 1110012L19Rik, Acsl4, G6pdx, Kif4, Ocrl, Pgk1, Phf8, Pja1, Prps2, Rbmx, Tmem185a, Vma21, Enox2
Cast-Monoallelic
F
% of overlap of XCR transcription and chromatin accessibility dynamics
XCR transcription dynamics (Janiszewski et al. 2019)
0 50 29 9 3 0 2 0 3 7 escapees
0 17 14 35 17 0 8 15 15 7 early
0 0 43 48 66 56 55 56 49 53 intermediate
0 17 14 9 11 44 33 27 31 13 late
0 0 0 0 3 0 2 2 3 20 very late
G
Enhancers
Zf
TEA
bZIP
E2F HTH bHLH ETS HMG Homeo box MADS
Promoters
bZIP bHLH Homeobox
% of TF Motif enrichment
H
OCT4
SOX2
KLF4
cMYC
ESRRB
PRDM14
NANOG
p300
sum score
I
Acot9
Chr X: 155262072-155263071
Sat1
Chr X: 155216220-155217173
Early 1 cluster region
ATAC-seq Day 8
RNA-seq Day 8
ChIP-seq OCT4 (ESCs)
Chronis et al. (2017)
OCT4 ChIP-qPCR region
Janiszewski et al. (2019)
Escapees 1
Escapees 2
Mus-Mono to Bi
Early 1
Early 2
Intermediate
Late 1
Late 2
Very late
Cast-Monoallelic

Fig S6

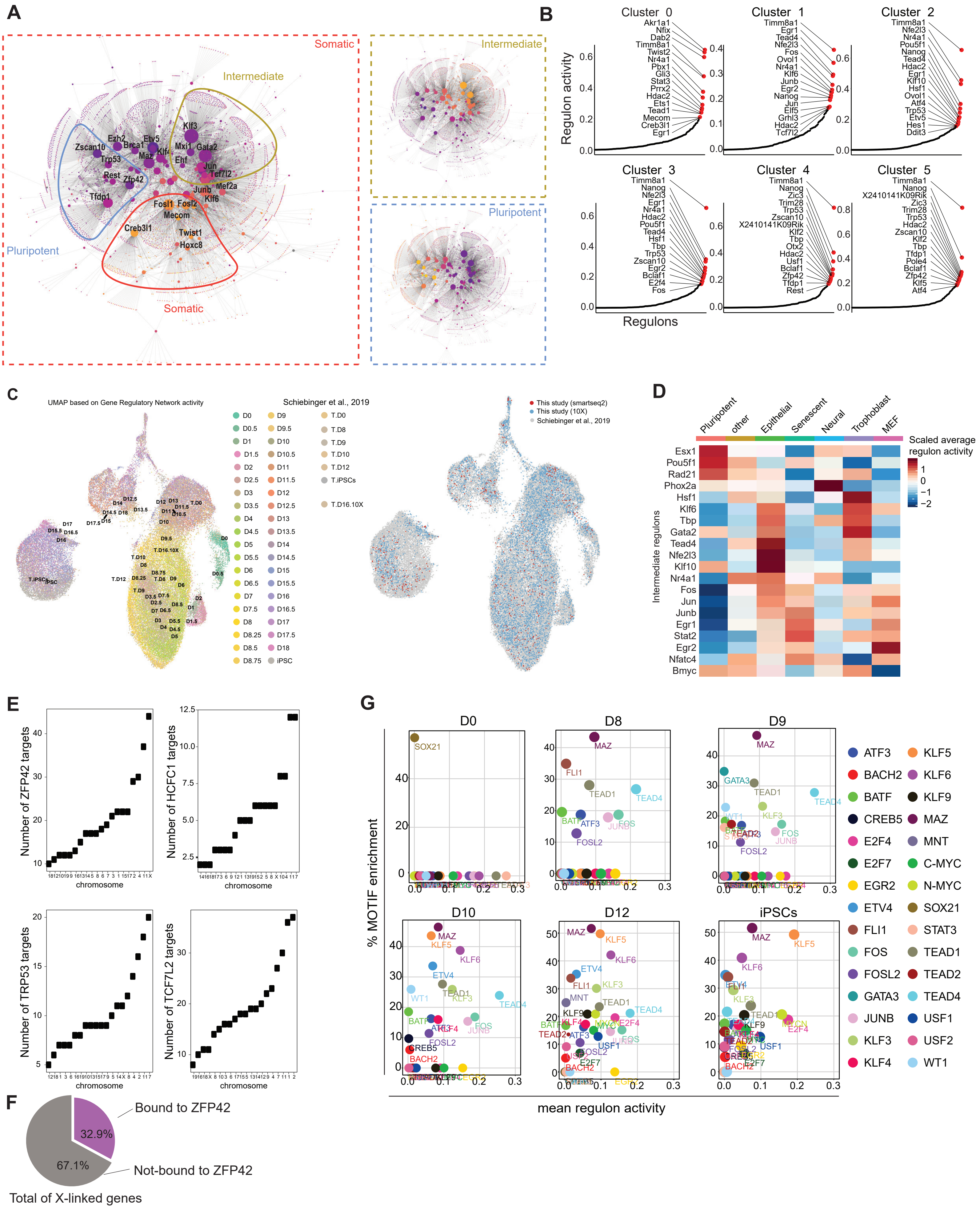
A
Somatic
Intermediate
Pluripotent
Klf3
Ezh2
Etv5
Zscan10
Brca1
Mxi1
Gata2
Klf4
Trp53
Maz
Ehf
Jun
Tcf7l2
Rest
Zfp42
Mef2a
Junb
Klf6
Tfdp1
Fosl1
Fosl2
Mecom
Creb3l1
Twist1
Hoxc8
B
Cluster 0
Cluster 1
Cluster 2
Cluster 3
Cluster 4
Cluster 5
Regulon activity
Regulons
C
UMAP based on Gene Regulatory Network activity
Schiebinger et al., 2019
This study (smartseq2)
This study (10X)
Schiebinger et al., 2019
D
Pluripotent
other
Epithelial
Senescent
Neural
Trophoblast
MEF
Scaled average regulon activity
Intermediate regulons
Esx1
Pou5f1
Rad21
Phox2a
Hsf1
Klf6
Tbp
Gata2
Tead4
Nfe2l3
Klf10
Nr4a1
Fos
Jun
Junb
Egr1
Stat2
Egr2
Nfatc4
Bmyc
E
Number of ZFP42 targets
Number of HCFC1 targets
Number of TRP53 targets
Number of TCF7L2 targets
chromosome
F
Bound to ZFP42
32.9%
67.1%
Not-bound to ZFP42
Total of X-linked genes
G
D0
D8
D9
D10
D12
iPSCs
% MOTIF enrichment
mean regulon activity
ATF3
BACH2
BATF
CREB5
E2F4
E2F7
EGR2
ETV4
FLI1
FOS
FOSL2
GATA3
JUNB
KLF3
KLF4
KLF5
KLF6
KLF9
MAZ
MNT
C-MYC
N-MYC
SOX21
STAT3
TEAD1
TEAD2
TEAD4
USF1
USF2
WT1
