# Supplementary material for: Enhanced chromatin accessibility contributes to X chromosome dosage compensation in mammals
Source: Genome Biol. 2021 Nov 1;22:302. doi: 10.1186/s13059-021-02518-5 (PMC8558763; doi:10.1186/s13059-021-02518-5)
Supplement: Supplementary file 3 — Additional file 3: Table S2. ChromHMM emission probabilities. Each row represents a distinct chromatin state based on the putative annotation. Cells show the modelled frequency of each histone mark in either ESCs or MEFs. [file 13059_2021_2518_MOESM3_ESM.pdf]

State	State annotation	MEF_H3K9ac	ESC_H3K9ac	MEF_H3K4me3	ESC_H3K4me3	MEF_H3K4me2	ESC_H3K4me2	MEF_H3K4me1	ESC_H3K4me1	MEF_H3K27ac	ESC_H3K27ac	MEF_H3K27me3	ESC_H3K27me3	MEF_H3K79me2	ESC_H3K79me2	MEF_H3K36me3	ESC_H3K36me3	MEF_H3K9me3	ESC_H3K9me3
1	Active promoters	98,07%	88,60%	91,76%	99,01%	91,70%	93,70%	27,95%	28,68%	97,51%	80,98%	1,52%	9,39%	93,38%	74,66%	5,60%	3,39%	0,93%	3,00%
2	Poised promoters	69,25%	91,37%	59,59%	98,94%	90,45%	88,30%	45,47%	28,09%	93,06%	66,92%	3,97%	10,57%	3,29%	6,35%	0,59%	0,01%	0,34%	2,61%
3	Inactive poised promoters	3,82%	78,76%	7,10%	78,32%	63,20%	97,45%	51,06%	43,39%	9,60%	14,31%	67,85%	65,62%	3,12%	1,49%	2,48%	0,67%	8,40%	8,82%
4	Enhancers	10,50%	3,74%	0,96%	0,00%	81,61%	1,30%	89,90%	3,22%	90,30%	0,80%	1,61%	4,86%	0,39%	0,22%	3,21%	0,74%	1,76%	3,49%
5		11,98%	34,94%	1,70%	7,86%	78,05%	82,50%	86,66%	81,20%	58,59%	38,57%	3,21%	3,92%	1,74%	2,56%	5,26%	0,85%	0,86%	2,92%
6		0,21%	19,97%	0,07%	0,61%	1,72%	92,89%	10,80%	93,30%	0,69%	70,70%	15,86%	0,52%	0,30%	0,99%	1,63%	1,30%	1,42%	6,25%
7		0,24%	1,45%	0,06%	0,00%	0,91%	8,43%	3,75%	57,36%	0,37%	17,72%	5,50%	0,55%	0,09%	0,26%	0,96%	3,15%	0,42%	2,04%
8		0,40%	69,84%	0,19%	57,69%	17,11%	99,64%	28,70%	76,88%	4,81%	85,56%	21,59%	1,07%	1,78%	39,38%	6,38%	5,53%	2,52%	6,83%
9	Poised enhancers	0,10%	25,70%	0,06%	0,80%	2,52%	64,22%	8,94%	68,41%	0,12%	7,13%	67,62%	14,18%	0,02%	0,55%	0,22%	0,43%	2,31%	2,93%
10		0,66%	6,53%	0,14%	0,00%	24,48%	1,59%	84,00%	1,81%	8,96%	0,37%	55,06%	12,59%	0,19%	0,09%	6,67%	0,24%	1,48%	5,74%
11	Transcribed enhancer	1,35%	29,30%	0,21%	8,27%	6,80%	85,74%	39,28%	86,02%	14,62%	55,98%	1,25%	0,20%	92,65%	85,94%	57,04%	23,58%	2,02%	2,62%
12		29,28%	33,49%	8,37%	26,23%	96,54%	88,61%	81,91%	86,22%	59,83%	46,37%	0,57%	2,14%	92,63%	85,54%	30,42%	12,95%	0,66%	1,58%
13		12,85%	9,21%	3,54%	0,08%	66,66%	4,86%	82,16%	9,77%	62,23%	2,35%	2,25%	7,41%	91,00%	25,42%	47,98%	5,16%	1,24%	2,05%
14	Gene body enhancers	2,98%	10,35%	0,32%	0,02%	23,15%	1,33%	81,42%	4,37%	45,72%	2,79%	5,99%	1,52%	3,56%	1,88%	82,11%	15,79%	1,37%	2,09%
15		0,57%	41,02%	0,09%	7,21%	0,48%	61,52%	14,78%	68,45%	8,50%	41,29%	2,27%	0,88%	3,92%	8,23%	87,70%	19,03%	0,60%	2,46%
16	Gene body	0,37%	4,86%	0,11%	0,00%	0,04%	0,28%	2,80%	1,34%	1,48%	0,30%	3,75%	1,20%	0,82%	0,12%	80,59%	8,07%	0,67%	1,32%
17		0,70%	3,86%	0,11%	0,01%	0,04%	0,03%	1,71%	3,00%	2,70%	2,84%	1,08%	0,01%	2,72%	4,64%	89,71%	67,70%	0,32%	1,20%
18	Transcribed regions	0,40%	8,25%	0,07%	0,33%	3,25%	13,71%	8,35%	32,32%	1,17%	10,89%	7,98%	0,17%	6,20%	67,82%	12,47%	20,83%	1,13%	3,63%
19		1,37%	8,00%	0,24%	0,26%	0,63%	1,19%	10,20%	16,77%	6,49%	9,44%	1,25%	0,03%	76,02%	71,78%	92,34%	68,23%	1,96%	3,70%
20		1,32%	4,12%	0,26%	0,01%	14,31%	8,37%	20,42%	31,43%	4,35%	2,61%	0,41%	0,06%	97,45%	90,34%	29,55%	7,29%	1,00%	0,88%
21		0,66%	1,36%	0,18%	0,00%	2,07%	0,73%	14,39%	3,14%	2,78%	0,27%	2,46%	1,46%	80,72%	6,32%	45,30%	3,39%	1,90%	1,85%
22	Repressed (Polycomb)	0,09%	1,73%	0,06%	0,00%	0,08%	0,11%	1,26%	0,54%	0,07%	0,12%	53,72%	5,63%	0,01%	0,05%	0,27%	0,28%	1,53%	2,51%
23		0,19%	1,67%	0,12%	0,02%	1,58%	1,09%	3,15%	1,50%	0,15%	0,05%	66,12%	69,94%	0,60%	0,06%	1,52%	0,70%	5,75%	5,13%
24	Repressed (Constitutive heterochromatin)	0,32%	1,01%	0,30%	1,04%	0,55%	1,22%	4,65%	2,09%	2,29%	9,07%	32,23%	5,85%	0,43%	0,75%	13,51%	17,72%	33,91%	63,51%
25		0,01%	0,09%	0,02%	0,05%	0,05%	0,17%	0,26%	0,10%	0,02%	0,38%	1,82%	1,00%	0,00%	0,00%	0,05%	4,45%	5,19%	49,82%
26	Low signal	0,02%	0,00%	0,03%	0,00%	0,05%	0,02%	0,04%	0,05%	0,01%	0,00%	0,20%	0,36%	0,00%	0,00%	0,01%	0,02%	0,13%	0,25%
27		0,27%	0,52%	0,09%	0,00%	0,23%	0,05%	1,87%	0,59%	0,19%	0,07%	0,91%	0,29%	0,86%	0,44%	11,06%	2,66%	0,26%	0,34%
28		0,14%	0,18%	0,08%	0,00%	0,07%	0,04%	0,70%	0,14%	0,06%	0,03%	5,90%	2,44%	0,00%	0,00%	0,21%	0,10%	0,46%	0,84%
29		1,27%	0,56%	0,24%	0,00%	36,85%	0,34%	47,19%	1,82%	9,17%	0,13%	0,01%	2,30%	0,21%	0,05%	0,75%	0,18%	0,70%	1,18%
30		0,13%	47,88%	0,04%	14,29%	3,29%	71,09%	11,93%	24,58%	0,70%	2,98%	4,65%	3,37%	0,15%	0,21%	1,20%	0,45%	1,02%	5,60%
