# Supplementary material for: Enhanced chromatin accessibility contributes to X chromosome dosage compensation in mammals
Source: Genome Biol. 2021 Nov 1;22:302. doi: 10.1186/s13059-021-02518-5 (PMC8558763; doi:10.1186/s13059-021-02518-5)
Supplement: Supplementary file 7 — Additional file 7. Review history [file 13059_2021_2518_MOESM7_ESM.docx]

**Review history**

**First round of review**

**Reviewer 1**

Talon & Janiszewski et al report a time-course study to examine X-chromosome dosage regulation during reprogramming to mouse iPSCs. This is a continuous study (Janiszewski et al 2019) on this subject with new allelic ATAC-seq and single-cell RNA-seq analyses. This study confirms results from their previous one and from others, and provides new insights of regulation on XCR and XCU. Intriguingly, it shows enhanced accessibility on the upregulated Xa, which is erased during reprogramming and companied with changes of transcriptional burst and expression downregulaton of the Xa. While this omics study suggests an epigenetic sensing mechanism for XCU, a functional study to dissect key factors involved in this process has not been explored. Clarification of a few questions below could strengthen this paper.

1. The GRN analysis provides candidate TFs for future functional studies of XCR. Is it possible to try this analysis on X-linked genes on the Xa during reprogramming and XCD?

2. The authors show enhanced transcriptional burst frequency but decreased burst size of the Xa in MEFs. This is different from Larsson et al., 2019, which shows enhanced burst frequency of the Xa but no difference of burst size compared to autosomes. This discrepancy needs to be discussed with some explanations. In addition, both burst frequency and size are positively related with gene expression levels. How these two opposite regulations (enhanced burst frequency but decreased burst size) lead to X upregulation needs to be discussed.

3. Why not show the transcriptional burst analysis in XO cells from scRNA-seq?

4. Figure 4: it is helpful to show boxplots of burst frequency and size of the Xa and each autosome for each cluster cells (C0-5); no X-Y axis labels for Fig. 4C; better use the same X-axis scales in compared panels in Fig. 4C

Minor points:

1. Fig. S1A: please describe what the X-linked gene regions represent.

2. Typos: Page 8, line 60, delete “given”; Page 10, line 45, “innaccessibility”; Page 16, line 28, “Di” should be “Nguyen”.

**Reviewer 2**

This interesting manuscript explores mammalian dosage compensation from the unique perspective of chromatin accessibility during reactivation of the inactive X during the reprogramming to pluripotency.

The paper is clearly written and the results generally logically presented.  I will present some issues to be addressed.  A couple of recurrent points are that in addition to p-values it would be helpful to include proportional changes, and that when previously published datasets were included, there could be more discussion of approaches and comparison of results of analyses (again proportional change, in addition to significance).

Introduction: The final thesis of a critical role for ZFP42 relies upon the evolution of the protein in placental mammals. Therefore, it would be worth comparing what is known about XCU in mammals (placental and marsupial).

Figure 1:  The allele-specific comparison is a strength, as comparison to total (ie diploid) would perhaps obscure the difference.  However, it raises the question as to whether CAST/Mus are the same for the X. While a reverse cross would be ideal, perhaps comparing total in different datasets (which then would not require skewed informative crosses) would be sufficient validation. In addition, it would be helpful to include the proportional difference Xa relative to autosomes.

Figure 2:  The use of diagrams throughout the manuscript is very helpful. In panel E, similar panels have been color-coded (blue/red) - why not here.  This analysis could also validate whether there is a difference between CAST and Mus? It does seem by eye that Cast might be reduced?

Figure 3:  For the Schiebinger data, what is the source (is it also Cast/Mus), this should be described.  

What genes comprise the 'pluripotency GRN' (only the ones shown in Figure 3D?

There seems to be a clear inflection point (approximately pseudotiime 16)
Is this true for more genes in the genome, or specific to those shown?  More discussion seems warranted.
It was not clear to me what normalized expression was normalized to?
GFP seems to reactivate faster than Xist silences - is this due to half-life of the RNAs?

Figure 4 (and accompanying text): For transcriptional burst frequency and size, a brief description of method rather than simply the reference would be helpful. Comparison of both significance and proportional change would be helpful.
The effect on burst frequency/size is subtle - would using a matched autosomal set clarify the impact? How much variation is there across the autosomes - is the X distinctive?

Figure 5: Panel C is very hard to follow. D is used for days, and C0-C5 clusters were defined in Figure 3. Any new clusters should be given new (and perhaps more intuitive?) descriptors.
In Panel E it was not clear what the enrichment was relative to - the legend says 'over the whole genome'

Figure 6 (and accompanying text): Regulon requires a clear definition relative to this study, and then for panel E, how regulon activity is defined (relative to what) needs to be clarified. In panel
what about MYC?  The enrichment of TF sites on X versus autosomes (as in S6E) would be interesting to compare for all TFs - could this be added to either 5E or 6C (or 6E)?

Figure 7:  Are the proportional changes diagrammed the same as shown in the earlier figures? This would be clearer if those changes were listed. Here they seem more dramatic.  

The inclusion of (only) ZFP42 in this diagram (and abstract) to me suggests a causative rather than correlative role.  I have suggested additional controls regarding relative abundance of other TFs. The statement that ZFP42 is 'of particular interest' is justifiable.  The X chromosome is evolutionarily distinct, and thus could the enrichment of genes for gametogenesis contribute to the ZFP42 enrichment? Is it possible to match autosomal genes with equivalent expression patterns?

**Authors’ response to reviewers**

# Point-by-point Response to the Reviewers Comments:

(Reviewers comments are in blue)

**Manuscript ID:** GBIO-D-21-00699

**Manuscript Title:** Enhanced Chromatin Accessibility Contributes to X-Chromosome Dosage Dosage Compensation in Mammals.

We would like to sincerely thank the reviewers for their constructive comments and helpful suggestions. We have been able to address most of the points raised in our revised manuscript. We feel that these changes have improved our paper. Below we provide a point-by-point rebuttal to all of the comments made by the reviewers.

**Reviewer 1** appreciated the novelty and interest of our study and she/he provided comments that aimed at clarifying a few points and strengthening the study. Comments from Reviewer 1 related mainly to transcription factors (TFs) potentially involved in X chromosome downregulation (XCD) during reprogramming and to the analysis of transcriptional burst frequency and burst size.

Talon & Janiszewski et al report a time-course study to examine X-chromosome dosage regulation during reprogramming to mouse iPSCs. This is a continuous study (Janiszewski et al 2019) on this subject with new allelic ATAC-seq and single-cell RNA-seq analyses. This study confirms results from their previous one and from others, and provides new insights of regulation on XCR and XCU. Intriguingly, it shows enhanced accessibility on the upregulated Xa, which is erased during reprogramming and companied with changes of transcriptional burst and expression downregulation of the Xa. While this omics study suggests an epigenetic sensing mechanism for XCU, a functional study to dissect key factors involved in this process has not been explored. Clarification of a few questions below could strengthen this paper.

We would like to thank the referee for his/her comments on the interest and novelty of our work, as well as for comments which have greatly helped to strengthen the manuscript.

1. The GRN analysis provides candidate TFs for future functional studies of XCR. Is it possible to try this analysis on X-linked genes on the Xa during reprogramming and XCD?

**Response 1.** We agree with the reviewer that the GRN data can be used for predicting TFs that might be implicated in Xa upregulation and XCD. We set out to search for transcriptional activators of the hyperactive Xa by looking for TFs 1) whose regulon activity follows the Xa pattern in reprogramming and 2) which have X-linked targets predicted by SCENIC.

First, we looked for TFs whose regulon activity is higher at the beginning of reprogramming (higher activity in MEFs, when XCU is present) and decrease upon reprogramming as XCD takes place (lower activity in iPSCs). We found that the activity of the regulons Junb and Fosl1 is highly correlated with the Xa status during reprogramming and XCD (**Figure R1**). Second, we confirmed that these TFs were predicted by SCENIC to target several X-linked genes (**Figure 6D**, **Supplementary Table 5**).

While useful, we are aware that this new analysis has several limitations. First, due to SCENIC constraints, this analysis is limited to transcriptional activators, therefore, we might miss transcriptional repressors that act to downregulate the hyperactive X during XCD. Second, we know that XCU is also present in XY mESCs (Larsson et al., 2019 *Nature Structural and Molecular Biology*) and XO iPSCs (**Figure 4**), therefore, other TFs might mediate XCU in the pluripotent stem cell state.

**Figure R1.** Relationship between regulon activity and Xa dynamics during reprogramming. Displayed are 2 regulons that are most predictive of Xa upregulation and its erasure (XCD) and which are also predicted to have >10 X-linked target genes. The fitted line on the plot was generated using loess function. Please note that the x axis represents regulon activity and not pseudotime, the latter is showed using a color gradient. The grey areas represent the 95% confidence interval. Regulon activity refers to the activity of a transcription factor, quantified by measuring the expression of the predicted target genes of a transcription factor.

2. The authors show enhanced transcriptional burst frequency but decreased burst size of the Xa in MEFs. This is different from Larsson et al., 2019, which shows enhanced burst frequency of the Xa but no difference of burst size compared to autosomes. This discrepancy needs to be discussed with some explanations.

**Response 2.** We have now discussed this point in the manuscript.

# Discussion section, page 19-20, lines 666-672:

*“Moreover, we also observed a reduced burst size on the Xa compared to the autosomes, which is contrary to what was found in a previous study [22], where autosomes and the upregulated Xa do not differ in transcriptional burst size. However, these different observations could arise due to the lack of unique molecular identifiers (UMIs) in the Smart-seq2 method used in our study. UMIs can help improve inference of transcriptional burst kinetics [67]. Therefore, it would be interesting to use*

*single-cell transcriptomics approaches that use UMIs, such as Smart-seq3, to further investigate this point.”*

In addition, both burst frequency and size are positively related with gene expression levels. How these two opposite regulations (enhanced burst frequency but decreased burst size) lead to X upregulation needs to be discussed.

**Response 3.** It is possible that increased burst frequency combined with decreased burst size could still lead to XCU if the increase in burst frequency leads to increased transcript production relative to the decreased production due to the decrease in burst size. In addition, it has been proposed that active transcriptional locations in the human genome have higher burst frequency but lower burst size than repressed locations (Zhao et al., 2021 *bioRxiv* https://doi.org/10.1101/2021.04.29.441875). Therefore, it could be that genes on the sole active X chromosome follow the modulation observed in most active genomic locations to upregulate their expression. However, we acknowledge that the differences in burst frequency and size between X-linked genes and autosomes observed in this study are small and that the discrepancy with previously published XCU results from (Larsson et al., 2019 *Nature Structural and Molecular Biology*) could be due to technical issues such as not using UMIs to infer transcriptional burst kinetics.

3. Why not show the transcriptional burst analysis in XO cells from scRNA-seq?

**Response 4.** We agree with the reviewer that showing transcriptional burst analysis in XO cells from scRNA-seq data is very interesting. We have now performed such transcriptional burst analysis on XO cells, specifically on *X-Cast O-Mus*, *X-Mus O-Cast* and *X-Cast X-Mus* iPSCs as a control. We found that, as expected, *X-Cast X-Mus* iPSCs with two active X chromosomes showed similar levels of burst frequency and size in both alleles for X-linked genes compared to autosomal genes (**Figure S4G**). *X-Cast O-Mus* iPSCs showed a significant increase in burst frequency on the *Cast* allele, in agreement with XCU, and no significant change in burst size, in agreement with the study of (Larsson et al., 2019 *Nature Structural and Molecular Biology*) (**Figure S4G**). These results are also observed when the opposite allele is lost (**Figure S4G**). Specifically, the *Mus* allele in *X-Mus O-Cast* showed increased burst frequency, again in agreement with previous work. Moreover, *X- Mus O-Cast* iPSCs displayed a significant decrease in burst size of the X chromosome compared to autosomes, which recapitulates our findings of transcriptional burst in *Xa-Cast Xi-Mus* (C0 cells) from **Figure 4C**. Taking these results together, although these analyses have been conducted with limited amount of cells (15 *X-Cast O-Mus* cells and 9 *X-Mus O-Cast* cells), burst size seems to be less sensitive to the X chromosome status than transcriptional burst frequency, the latter changes more abruptly depending on the X chromosome status, as observed in (Larsson et al., 2019 *Nature Structural and Molecular Biology*).

We have now added these results in **Figure S4G**, and in the manuscript.

# Results section, page 11, lines 351-368:

*“Moreover, we asked if X-chromosome loss results in changes in transcriptional burst frequency and size in iPSCs. We found that XX iPSCs showed similar levels of burst frequency and size on both alleles of the X chromosome and on autosomes (Figure S4G, top row). Cells which lost an X chromosome (X- Cast O-Mus) showed a significant increase in burst frequency of X-linked genes and no decrease in burst size in agreement with XCU and in agreement with a previous study [22] (Figure S4G, middle row). Burst frequency was also increased when the opposite X-chromosome allele is lost. Specifically, the cells which lost the Cast X chromosome (X-Mus O-Cast) displayed increased burst frequency of X- linked genes (Figure S4G, bottom row), and a significant decrease in burst size compared to autosomes (Figure S4G, bottom row), which recapitulates our findings of transcriptional burst in MEFs (Figure 4C). As expected, X-chromosome loss was apparent from the depletion of reads from the aneuploid X chromosome (Figure S4G). Therefore, XO cells undergo changes in transcriptional burst on their sole X chromosome that might contribute to XCU.*

*Collectively, the results show that XCU is erased concomitant with XCR during reprogramming, then reinstated after X-chromosome loss in female iPSCs and accompanied by an increase in transcriptional burst frequency. X-chromosome dosage sensing and compensation are thus intrinsic properties of cells both in the differentiated and undifferentiated states.”*

# Figure legends section, page 48, lines 1715-1717:

*“G. Distribution of burst frequency and burst size of autosomal and X-linked genes on the Cast allele in X-Cast O-Mus, X-Mus O-Cast and X-Cast X-Mus iPSCs. A Wilcoxon rank-sum test was used for significance testing.”*

4. Figure 4: it is helpful to show boxplots of burst frequency and size of the Xa and each autosome for each cluster cells (C0-5); no X-Y axis labels for Fig. 4C; better use the same X-axis scales in compared panels in Fig. 4C.

**Response 5.** As suggested, we have now added boxplots of burst frequency and size of the Xa chromosome and each autosome in the *Cast* allele for clusters C0 to C5 (**Figure S4D**). We have also added the X-Y axis labels for **Figure 4C** and used the same axis scales for the panels that are compared in this figure.

# Figure legends section, page 48, lines 1706-1707:

*“D. Box plots with burst frequency and burst size for all chromosomes in C0-C5 cluster cells in the Cast allele.”*

Minor points:

1. Fig. S1A: please describe what the X-linked gene regions represent.

**Response 6.** We have now added the following information to **Figure S1A** to better describe what the X-linked gene regions represent:

*Col1a1*: Exon 19-20

*Acot9*: Exon 1

*Enox2*: Exon 1

*Kdm6a*: Exon 1

2. Typos: Page 8, line 60, delete “given”; Page 10, line 45, “innaccessibility”; Page 16, line 28, “Di” should be “Nguyen”.

**Response 7.** We have now corrected these typos.

**Reviewer 2** found our manuscript interesting, clearly written and with well-presented results and made useful suggestions. We are grateful to the reviewer for the detailed analysis of the manuscript and for the excellent suggestions that have helped to improve the study.

This interesting manuscript explores mammalian dosage compensation from the unique perspective of chromatin accessibility during reactivation of the inactive X during the reprogramming to pluripotency.

The paper is clearly written and the results generally logically presented. I will present some issues to be addressed.

A couple of recurrent points are that in addition to p-values it would be helpful to include proportional changes, and that when previously published datasets were included, there could be more discussion of approaches and comparison of results of analyses (again proportional change, in addition to significance).

**Response 1.** We agree with the reviewer that proportional changes, in addition to p-values, will make the results easier to interpret. We have now added proportional changes to several results obtained with our data and with published datasets throughout the manuscript, now included in **Figure 1D, S2I** and **4A**, and/or in the manuscript text.

# Results section, page 5, lines 165-169:

*“Specifically, the Xa was globally 1.33 times more accessible than the median of all autosomes on the same allele (Figure 1D, right). These results suggest that a 1.33 fold increase in chromatin accessibility contributes to X-chromosome to autosome gene dosage compensation, which is in line with what is observed at the transcriptional level [22].”*

# Results section, page 6-7, lines 208-211:

*“While both X chromosome alleles in female mESCs showed the same level of accessibility as autosomes, resembling the X chromosome state of XX iPSCs (Figure 2E-F), we found that chromatin accessibility on the X-Mus chromosome of male XY mESCs was increased 1.4 fold over that of autosomes (Figure S2I-J).”*

# Results section, page 9, lines 296-299:

*“Unexpectedly, we found that XCD, the erasure of XCU on the Xa of MEFs, took place during iPSC reprogramming. The median expression of X-Cast genes was 1.35 fold higher than that of autosomes in MEFs (C0), while it was below that of autosomes in iPSCs (C5) (Figure 4A, S4A).”*

# Results section, page 10, lines 326-331:

*“We found that burst frequency was indeed significantly increased (1.19 fold increase) on the Xa relative to autosomes in MEFs (Figure 4C, left, cluster C0, Figure S4D), in line with previous reports in mouse primary fibroblasts [22]. However, we also observed lowered burst size (0.82 fold decrease) on the Xa relative to autosomes in MEFs (Figure 4C, right, Figure S4D), which is not concordant with previous reports in mouse primary fibroblasts [22].”*

We hope that these analyses address the reviewer’s question.

Introduction: The final thesis of a critical role for ZFP42 relies upon the evolution of the protein in placental mammals. Therefore, it would be worth comparing what is known about XCU in mammals (placental and marsupial).

**Response 2.** As suggested by the reviewer, we have now discussed, in the introduction of the manuscript, what is known about XCU in mammals, including placental (mice, non-human primates and humans) and marsupial mammals. Briefly, female marsupials show imprinted XCI on the paternal X, and XCU on the remaining X chromosome. Male marsupials also show XCU on their sole Xa. XCU starts very early in development (E3.5). This upregulated state of the X chromosome is maintained through early development, including in the pluripotent epiblast, and in the adult organism, indicating that pluripotency is uncoupled from the X chromosome state in these mammals.

# Introduction section, page 3, lines 75-77:

*“XCU has been reported in several placental mammals including mice [18,22], marmosets (non- human primates) [23], humans [18], and in non-placental mammals such as marsupials [14].”*

# Page 3, lines 86-88:

*“In mice and marsupials, XCU is initiated within the first 3-4 days of development, when imprinted XCI is also initiated in females. XCU is also observed on the sole Xa in males [14,24,34–36].”*

# Page 3, lines 101-103:

*“In mice, humans and marsupials, XCR takes place in vivo in female primordial germ cells (PGCs) [35,40,41], and in the naive mouse epiblast, with the exception of marsupials where XCR does not take place in the epiblast [14].”*

Figure 1: The allele-specific comparison is a strength, as comparison to total (ie diploid) would perhaps obscure the difference. However, it raises the question as to whether Cast/Mus are the same for the X. While a reverse cross would be ideal, perhaps comparing total in different datasets (which then would not require skewed informative crosses) would be sufficient validation.

**Response 3.** We agree with the reviewer that it would be interesting to know if both *Cast* and *Mus* alleles are subject to hyperaccessibility. We have made new analyses that suggest that hyperaccessibility can take place on the X-*Cast* or X-*Mus*. While we do not have data to perform the same analysis with MEFs which have a Xa*-Mus*, we were able to reanalyze published ATAC- seq data from XY mESCs with a Xa*-Mus* (Song et al., 2019 *Stem Cell Reports*). We now show in **Figure S2J**, that the Xa*-Mus* allele can also be subjected to hyperaccessibility when present as a single active copy. This is apparent by comparing the level of accessibility of the sole Xa*-Mus* of male XY mESCs to that of the autosomes (**Figure S2J**). Therefore, both the X*-Cast* or X-*Mus* can be subject to increased chromatin accessibility. This is in agreement with our finding that the X*-Mus* or X*-Cast* are transcriptionally upregulated after loss one of the two X chromosomes in iPSCs (**Figure 4**). In addition, we have added proportional differences in accessibility of the X chromosomes relative to autosomes for the (Song et al., 2019 *Stem Cell Reports*) dataset (**Figure S2I**) and our study data (**Figure 1C, D**). We observed in our XX MEFs with one Xi and one hyperaccessible Xa and in XY mESCs with one hyperaccessible Xa that the level of hyperaccessibility of the sole Xa in both female and male cells is increased by 1.3-1.4 fold over the global level of autosomal accessibility (**Figure 1D, S2I**). This level of hyperaccessibility of the Xa compared to autosomes is in line with our results for transcriptional XCU (**Figure 4A**) and with the transcriptional study from (Larsson et al., 2019 *Nature Structural and Molecular Biology*).

Therefore, the single Xa in mouse cells can show enhanced chromatin accessibility, regardless of genetic background.

In addition, it would be helpful to include the proportional difference Xa relative to autosomes. This comment has been addressed above in the Reviewer 2, Response 1.

Figure 2: The use of diagrams throughout the manuscript is very helpful. In panel E, similar panels have been color-coded (blue/red) - why not here. This analysis could also validate whether there is a difference between CAST and Mus? It does seem by eye that Cast might be reduced?

**Response 4.** We are happy the reviewer found the diagrams helpful. We agree with the reviewer that preserving a color-code for *Mus/Cast* (Blue/Red) alleles would be helpful. As suggested, we have colored the *Mus* allele in blue and the *Cast* allele in red in **Figure 2** (Panel 2E-F, S2I-K) to follow the same color code as presented in **Figure 1** and in the rest of **Figure 2**.

It is true that in **Figure 2E** (current **Figure 2F**), some of the X*-Cast* chromatin accessibility regions shown seem to be reduced in accessibility in XX mESCs (CM1 and CM2) (reanalysis from Song et al., 2019 *Stem Cell Reports*). To assess if this is observed at a global level, we have now analyzed global levels of accessibility in both *Cast* and *Mus* alleles from XX mESCs (Song et al., 2019 *Stem Cell Reports*). We now show in **Figure 2E** that Xa*-Mus* and Xa*-Cast* in the mESC lines analyzed show very similar global levels of accessibility with no significant differences between them. Therefore, the *Cast* allele is not globally differentially accessible compared to the *Mus* allele in CM1 and CM2 mESC lines (**Figure 2E-F**) and has comparable levels of accessibility as their autosomes counterparts in these cell lines (**Figure S2I**).

# Results section, page 6-7, lines 208-211:

*“While both X chromosome alleles in female mESCs showed the same level of accessibility as autosomes, resembling the X chromosome state of XX iPSCs (Figure 2E-F), we found that chromatin accessibility on the X-Mus chromosome of male XY mESCs was increased 1.4 fold over that of autosomes (Figure S2I-J).”*

Figure 3: For the Schiebinger data, what is the source (is it also Cast/Mus), this should be described.

**Response 5.** We apologize we did not make this clearer in the original submission. The cells analyzed from the reprogramming study of (Schiebinger et al., 2019 *Cell*) come from a *Mus* genetic background with no *Cast* background, which does not allow for allele-resolution analyses.

We have added this information to the manuscript, in the results and methods section.

# Results section, page 8, lines 250-252:

*“We also used a published single-cell atlas of iPSC reprogramming with Mus genetic background, which does not enable allele-resolution analyses [59].”*

# Methods section, page 27, lines 960:

*“with Mus background,”*

What genes comprise the 'pluripotency GRN' (only the ones shown in Figure 3D?

**Response 6.** The ‘pluripotency GRN’ contains all of the pluripotency genes detected in this study as well as their predicted target genes that are also expressed in pluripotent stem cells. In **Figure 3D**, we showed a representative subset of these genes that are activated at different times during the reprogramming timecourse. For instance, *Zfp42* and *Pecam1* are activated early in reprogramming, followed by *Nanog*, *Tet1* and *Esrrb* (activated at mid reprogramming pseudotime) and *Dppa3*, *Dppa4* and *Prdm14* which are activated later in reprogramming. We have clarified this in the figure legend of **Figure 3D**.

# Figure legends section, page 43, lines 1519:

*“D. Normalized expression levels of representative pluripotency markers genes plotted along pseudotime…”*

*There seems to be a clear inflection point (approximately pseudotime 16). Is this true for more genes in the genome, or specific to those shown? More discussion seems warranted.*

**Response 7.** We agree with the reviewer that there is an inflection point during reprogramming pseudotime 16 for some of the genes including *Zfp42* and *Pecam1* (**Figure 3D**). We believe this inflection point corresponds to the mesenchymal-to-epithelial transition that takes place during reprogramming, and is one of the intermediate steps during iPSC reprogramming from MEFs, as defined in (Li et al. 2010 *Cell Stem Cell*; Samavarchi-Therani 2010 *Cell Stem Cell*, as well as Pasque et al., 2014 *Cell*; Schiebinger et al., 2019 *Cell*). This is well apparent also in our study, where other genes, including epithelial markers, start to be expressed in C1 and C2 cells at reprogramming pseudotime 16, such as *Cdh1* or *Epcam* (**Figure 3F**). Therefore, more genes in the genome, such as the ones shown in **Figure 3F** in C1, but also others, increase their expression around pseudotime 16, which coincides with the mesenchymal-to-epithelial transition, one of the first steps towards successful iPSC reprogramming.

We have now revised the manuscript accordingly:

# Results section, page 7, lines 233-236:

*“As expected, we detected the activation of pluripotency-associated genes including the early activation of Pecam1 and Zfp42, followed by Nanog, Tet1 and Esrrb, and others later in reprogramming such as Dppa3/4 and Prdm14 (Figure 3D).”*

# Results section, page 7-8, lines 239-245:

*“We detected a gradual switch from a MEF-specific state to a mesenchymal-to-epithelial transition state (C1 cells), which is one of the first step towards successful reprogramming (Figure 3E-F) [60,61] and is followed by a proliferative state and the activation of early pluripotency markers such as Zfp42, Pecam1 and Nanog (C2 cells, Figure 3D-F). This wave of transcriptional activation is followed by the acquisition of additional pluripotency genes, including Dppa4 and Dppa5a (C4 and C5 cells), as expected [58,59,62–64] (Figure 3E-F, S3B).”*

It was not clear to me what normalized expression was normalized to?

**Response 8.** We apologize this was not clear. We have now clarified that gene expression was normalized to library size (number of total counts per library) in the legend of **Figure 3**.

# Figure legends section, page 43, lines 1543-1544:

*“Gene expression levels were normalized to library size (number of total counts per library) in D, F, H and I.”*

GFP seems to reactivate faster than Xist silences - is this due to half-life of the RNAs?

**Response 9.** We thank the reviewer for this comment. We have analyzed studies of RNA half-life. In this study, we show that a small set of X-linked genes start reactivation before *Xist* has been completely silenced, but only reach full reactivation after *Xist* silencing. Therefore, these genes might be more dependent on *Xist* for the maintenance of their silencing. Most X-linked genes (termed intermediate and late genes), such as our GFP reporter, reactivate after *Xist* has started to decrease its expression, the pluripotent GRN is activated and repressive chromatin marks and factors are lost, such as HDACs, whose inhibition delayed the expression of GFP during XCR in our system (Janiszewski et al., 2019 *Genome Research*). Taking into account that *Xist* half-life is 20.22h (Tani et al., 2015 *Genome Biology* https://doi.org/10.1007/978-1-4939-2253-6_19), which is much longer than the average half-life of mouse transcripts (7.55h), we think that indeed it is possible that it takes longer for all *Xist* transcripts to be lost after *Xist* is silenced. Therefore, as suggested by the reviewer, the longer half-life of *Xist* could indeed account for the delay in complete *Xist* RNA loss during reprogramming compared to the reactivation of X-linked genes.

Figure 4 (and accompanying text): For transcriptional burst frequency and size, a brief description of method rather than simply the reference would be helpful. Comparison of both significance and proportional change would be helpful.

**Response 10.** We included a brief description of the methods used in our manuscript, as well as a methods section for the transcriptional burst analysis.

# Results section, page 10, lines 319-326:

*“Previous studies proposed that XCU in somatic cells is mediated by increased transcriptional burst frequency (how often a pulse of transcripts production occurs) [22,24]. We asked whether XCD during reprogramming could be mediated by changes in transcriptional burst frequency and/or burst size (number of transcripts generated per transcriptional pulse). We inferred transcriptional burst parameters with the two-state model of transcription, in which switching between ON and OFF states of a gene occurs at rates of kon and koff, and transcription only occurs in the ON state with a rate of ksyn [67]. This model provides allele-specific estimates of burst frequency (kon) and burst size (ksyn/koff) [22,24,67].”*

# Methods section, page 26, lines 911-920

*“Transcriptional burst analysis was performed as in [22,67]. Briefly, we normalized raw allelic counts using RPKM (Reads Per Kilobase Million) method. To differentiate missing data (NaN) from not expressed genes, we annotated missing allelic data (genes with expression but no allelic reads) to NaN and genes without expression to 0. Next, we estimated parameters of bursting kinetics with the publicly available function txburstML.py from (https://github.com/sandberg-lab/txburst) [67]. This function infers transcriptional burst parameters with the two-state model of transcription, as described in results section, toestimate burst frequency (kon) and burst size (ksyn/koff) [22,67]. Plots were performed with seaborn (v0.10.0) [115]. Wilcoxon rank-sum statistic was conducted for significant testing with scipy (v1.2.1) with the function scipy.stats.ranksums.”*

In addition, we have added proportional changes to our results in burst frequency and size in C0 (MEFs), which was also already addressed in **Reviewer 2, Response 1**.

The effect on burst frequency/size is subtle - would using a matched autosomal set clarify the impact?

**Response 11.** We agree with the reviewer that the effect on burst frequency and size is subtle. Therefore, following the reviewer’s suggestion, we have calculated the difference in burst frequency and size in C0 and C5 cells for the same number of randomly selected genes in autosomes as genes for which we had information on the X chromosome (227 genes for C0 and 266 genes for C5). In this analysis, we observed a significant increase in burst frequency and a significant decrease in burst size on the X-linked set of genes compared to the matched autosomal set in C0 but not C5 (**Figure R2**). These results are in line with our previous observations in **Figure 4C**.

**Figure R2.** Distribution of burst frequency and burst size in C0 and C5 cells for the same number of randomly selected genes in autosomes and on the X chromosome on the *Cast* allele. A Wilcoxon rank-sum test was used for significance testing.

How much variation is there across the autosomes - is the X distinctive?

**Response 12.** We have looked at the autosomes and the X chromosome variation in burst frequency and size for the clusters C0 to C5, now addressed in **Figure S4D**. As shown in **Figure S4D**, despite variation on autosomes, the X chromosome appears to be distinctive from the autosomes in C0 in burst frequency and size, this difference is erased in C1 to C5.

# Figure legends section, page 48, lines 1706-1707:

*“D. Box plots with burst frequency and burst size for all chromosomes in each XCR cluster (C0-C5) in the Cast allele.”*

Figure 5: Panel C is very hard to follow. D is used for days, and C0-C5 clusters were defined in Figure 3. Any new clusters should be given new (and perhaps more intuitive?) descriptors.

**Response 13.** We gave new intuitive names to the chromatin accessibility clusters and changed those in the corresponding **Figure 5** panels and in the text.

The new cluster names are now the following:

C7 has been changed to “Mus-Mono to Bi cluster”; C6 has been changed to “Escapees 1 cluster”;

C1 has been changed to “Escapees 2 cluster”;

C8 has been changed to “Early 1 cluster”; C5 has been changed to “Early 2 cluster”;

C3 has been changed to “Intermediate cluster”; C9 has been changed to “Late 1 cluster”;

C2 has been changed to “Late 2 cluster”; C10 has been changed to “Very late cluster” and

C4 has been changed to “Cast-Monoallelic cluster”.

# Results section, page 12, lines 389-396:

*“Inaccessible regions on the Xi-Mus that become biallelically accessible at day 8 (Early 1 and 2 clusters), day 9 (Intermediate cluster), day 10 (Late 1 and Late 2 clusters) and at day 12 (Very late cluster) during XCR, regions that remain biallelically accessible throughout reprogramming (Escapees 1 and Escapees 2 clusters), regions accessible from the Mus allele that become biallelically accessible (Mus-Mono to Bi cluster) and regions that are only accessible from the Cast allele (Cast- Monoallelic cluster) (Figures 5C, S5C).”*

In Panel E it was not clear what the enrichment was relative to - the legend says 'over the whole genome'.

**Response 14.** We performed motif enrichment analysis of X-linked regions that are biallelic at the different reprogramming time points compared to 50 000 random background genome regions. In this way, we identified motifs that are differentially enriched in biallelically accessible regions at a given time point compared to the random set of regions in the mouse genome. We now specified this in **Figure 5E** and **S5G** legends.

# Figure legends section, page 44, lines 1577-1578 and page 49, lines 1747-1748:

*“The color gradient represents the percentage of regions with enriched motifs in the indicated group over 50000 random background genome regions.”*

Figure 6 (and accompanying text): Regulon requires a clear definition relative to this study, and then for panel E, how regulon activity is defined (relative to what) needs to be clarified.

**Response 15.** As suggested, we have now included a clearer definition of regulon in the context of this study in the text manuscript (indicated below).

Results section, page 13-14, lines 452-458:

*“SCENIC first identifies TF targets based on gene co-expression with TFs in the same cell, then the list of targets is filtered to keep only the targets which contain a binding motif for a given TF [77]. The outcome is a list of regulons, where each regulon is a collection of predicted gene targets for a given TF. SCENIC identified 311 regulons active during reprogramming (Supplementary Table 3). Based on the activity of target genes, these regulons are predicted to be potentially involved in reprogramming.”*

Moreover, regulon activity refers to the proportion of expressed genes in the signature (regulon) and their relative expression values compared to the other genes within the cell. This definition has now also been added to the manuscript.

# Results section, page 14, lines 470-472:

*“Regulon activity can serve as a useful metric of TF activity and refers to the proportion of expressed genes in the signature (regulon) and their relative expression values compared to the other genes within the cell.”*

What about MYC? The enrichment of TF sites on X versus autosomes (as in S6E) would be interesting to compare for all TFs - could this be added to either 5E or 6C (or 6E)?

**Response 16.** As requested by the reviewer, we show below the number of predicted targeted genes in each chromosome for MYC (**Figure R3**). In addition, we have also determined the number of target genes per chromosome for all TFs. These analyses have now been included in **Supplementary Table 5**.

**Figure R3.** Number of predicted MYC targets on all chromosomes with SCENIC. In addition, we have added a representative subset of TFs with their number of targets on the X chromosome and on autosomes to **Figure S6E**. For this, we have chosen ZFP42, HCFC1 and TRP53, whose regulon activity is correlated with reprogramming pseudotime and XCR (top 3 regulons with highest correlation which also have a high number of X-linked targets predicted by SCENIC) (**Figure 6E**) and TCF7L1, whose regulon activity is anti-correlated with reprogramming pseudotime and XCR (**Figure 6E**).

# Results section, page 16, lines 531-536:

*“Moreover, HCFC1 and TRP53, whose regulon activity is correlated with reprogramming pseudotime and XCR (Figure 6E), also showed putative X-linked targets enriched on the X chromosome relative to autosomes (Figure S6E, Supplementary Table 5), contrary to TCF7L1 which has more putative targets enriched on autosomes (Figure S6E, Supplementary Table 5) and whose regulon activity is anti-correlated with reprogramming pseudotime and XCR (Figure 6E).”*

# Figure legends section, page 50, lines 1776-1777:

*“E. Number of ZFP42, HCFC1, TRP53 and TCF7L2 predicted targets on all chromosomes with SCENIC.”*

# Figure legends section, page 51, lines 1796-1797:

*“Supplementary Table 5. Numbers of Regulon targets per chromosome for all active Regulons during reprogramming.”*

Figure 7: Are the proportional changes diagrammed the same as shown in the earlier figures? This would be clearer if those changes were listed. Here they seem more dramatic.

**Response 17.** We carefully revised our **Figure 7** schematic model and made sure that the levels of chromatin accessibility and expression recapitulate the data showed in this manuscript. For both chromatin and expression, the hyperaccessible/upregulated X chromosome is between 1.3/1.4 times more accessible or transcriptionally upregulated than the single autosome level, the latter was set to 1 in **Figure 7**. When X chromosome hyperaccessibility/transcriptional upregulation is lost due to XCD, the X chromosomes acquire a similar level of accessibility and expression as those found in autosomes (level = 1). On the other hand, XY or XO cells present 1.3/1.4 times more accessibility and gene expression on their sole Xa compared to a single autosome (level 1). We think this is well represented in the **Figure 7**. We hope that the proportional changes now added to **Figures 1, 2** and **4** help clarify our point.

The inclusion of (only) ZFP42 in this diagram (and abstract) to me suggests a causative rather than correlative role. I have suggested additional controls regarding relative abundance of other TFs. The statement that ZFP42 is 'of particular interest' is justifiable. The X chromosome is evolutionarily distinct, and thus could the enrichment of genes for gametogenesis contribute to the ZFP42 enrichment?

**Response 18.** To answer this question, we have calculated the proportion of genes that are predicted as ZFP42 targets by SCENIC that are related to gametogenesis versus those that are not related to gametogenesis in each autosome and on the X chromosome. For this comparison, we have used genes involved in gametogenesis, the genes associated to the biological process GO term “gamete generation” in *Mus musculus* (GO:0007276). Here, we found that Zfp42 regulon targets on the X chromosome are not highly enriched for gametogenesis genes (only 4.5% of *Zfp42* targets are genes involved in gamete formation) (**Figure R4**). In fact, there are other autosomes which have a higher proportion of genes involved in gametogenesis and that are predicted to be Zfp42 targets, such as chromosomes 2, 3, 4, 5, 6, 8, 9, 12, 13 and 16 (**Figure R4**). Moreover, we quantified the percentage of genes involved in gametogenesis that belong to each autosome and the X chromosome (not necessarily ZFP42 targets) and we did not observe an increased proportion of gametogenesis genes on the X chromosome compared to the autosomes (**Figure R5**). Therefore, the enrichment of gametogenesis genes on the X chromosome does not seem to be related with the higher predicted SCENIC targets on the X chromosome compared to other autosomes for ZFP42.

**Figure R4.** The percentage of ZFP42 target genes predicted by SCENIC that are (pink) or are not (grey) involved in gametogenesis among all autosomes and the X chromosome.

**Figure R5.** The percentage of genes involved in gametogenesis that belong to each autosome and the X chromosome.

Is it possible to match autosomal genes with equivalent expression patterns [to ZFP42]?

**Response 19.** As suggested by the reviewer, we searched for autosomal genes with equivalent gene expression patterns to *Zfp42* during reprogramming. To do so, we selected the most variable genes during reprogramming and filtered out those genes with clearly inequivalent expression patterns to *Zfp42*, such as genes highly expressed in MEFs or not expressed in iPSCs. After performing k-means clustering with the resulting set of genes, we kept the autosomal genes which clustered together with *Zfp42*, indicating that those autosomal genes have similar levels of expression as *Zfp42* during iPSC reprogramming. In summary, we could identify 12 autosomal genes (*Bub1, Dhx16, Fbxo15, Gdf3, Hells, Jarid2, Mov10, Mtf2, Pigp, Tex19.1, Trap1a* and *Zscan10*) which have an equivalent gene expression pattern to *Zfp42* (**Figure R6**).

**Figure R6.** Expression of autosomal genes which follow *Zfp42* expression patterns during reprogramming plotted along pseudotime trajectory. Fitted line derived using loess function. Gene expression levels were normalized to library size.

**Second round of review**

**Reviewer 1**

The revision has well addressed most of the reviewers' comments.

**Reviewer 2**

The authors carefully and fully addressed both reviewers comments.
